# Self-Monitoring Theranostic Nanomaterials for Biomedical Imaging and Cancer Therapy: Real-Time Visualization of Biodistribution, Payload Release, and Therapeutic Response

**DOI:** 10.3390/nano16201309

**Published:** 2026-10-16

**Authors:** Chanju Choi, Dongseong Seo, Jonghyun Park, Dongmin Yu, Sihyun Song, Sohyeon Yu, Jeongmin Shin, Simseok A. Yuk, Daekyung Sung, Hyungjun Kim

**Affiliations:** 1 Department of Chemistry and Bioscience, Kumoh National Institute of Technology, 61 Daehak-ro, Gumi 39177, Republic of Korea; ccj2001@kumoh.ac.kr (C.C.); sjqhek123@kumoh.ac.kr (J.P.); mustdoing2816@kumoh.ac.kr (D.Y.); apple59@kumoh.ac.kr (S.S.); 2 Center for Bio-Healthcare Materials, Bio-Convergence Materials R&D Division, Korea Institute of Ceramic Engineering and Technology, 202 Osongsaengmyeong 1-ro, Osong-eup, Heungdeok-gu, Cheongju 28160, Republic of Korea; ds950826@gmail.com (D.S.); aelnlouis@yonsei.ac.kr (S.Y.); minnn3926@gmail.com (J.S.); 3 Department of Applied Bioengineering, Graduate School of Convergence Science and Technology, Seoul National University, Seoul 08826, Republic of Korea; 4 Department of Chemical and Biomolecular Engineering, Yonsei University, 50 Yonsei-ro, Seodaemun-gu, Seoul 03722, Republic of Korea; 5 Department of Biomedical Engineering, Northwestern University, Evanston, IL 60208, USA; andrewsim89@gmail.com

**Keywords:** self-monitoring nanomaterials, theranostic nanomedicine, biomedical imaging, cancer therapy, payload release, therapeutic response

## Abstract

Theranostic nanomedicines integrate therapeutic delivery with diagnostic imaging, but many conventional platforms primarily visualize nanocarrier localization and provide limited information on whether the therapeutic payload has been released, the intended treatment mechanism has been activated, or a meaningful biological response has occurred. Self-monitoring theranostic nanomedicines have emerged as functionally informative platforms capable of reporting their own biodistribution, biological fate, payload release, therapeutic activation, and downstream response in real time or through longitudinal imaging. This review summarizes the conceptual framework and design principles of self-monitoring nanomedicine, including always-on and activatable probes, Förster resonance energy transfer, self-quenching and dequenching mechanisms, ratiometric sensing, environment-responsive contrast agents, and multimodal imaging. Major imaging modalities, including fluorescence, magnetic resonance, photoacoustics, positron emission tomography, single-photon emission computed tomography, computed tomography, and ultrasound imaging, are discussed with emphasis on their respective capabilities and limitations for monitoring nanomedicine performance. Representative studies demonstrate the visualization of systemic biodistribution, target-site accumulation, carrier disassembly, drug and nucleic-acid release, photothermal and photodynamic activation, gene and RNA activity, oxidative stress, immune modulation, apoptosis, and pathogen clearance. Particular emphasis is placed on cancer theranostics, where self-monitoring nanomaterials can integrate tumor imaging with the assessment of intratumoral delivery, payload release, therapeutic activation, and early treatment response. Broader applications in inflammatory and immune-related diseases and infectious diseases further illustrate the general applicability of these monitoring principles beyond cancer. Despite substantial progress, clinical translation remains limited by ambiguous signal interpretation, insufficient quantitative validation, biological safety concerns, manufacturing complexity, and regulatory challenges. Future development should prioritize mechanistically defined and clinically actionable signals, orthogonal validation, standardized reporting, biodegradable and clearable materials, reproducible manufacturing, and compatibility with established imaging workflows. Self-monitoring theranostic nanomedicines may ultimately enable the transition from image-guided drug delivery to feedback-guided, adaptive, and personalized precision therapy.

## 1. Introduction

Nanomedicine has significantly reshaped the landscape of drug delivery by enabling the rational design of nanoscale systems capable of improving the pharmacokinetics, biodistribution, stability, and target-site accumulation of therapeutic agents [1]. Conventional nanocarriers, including liposomes, polymeric nanoparticles, micelles, dendrimers, inorganic nanoparticles, and biomimetic vesicles, have been extensively investigated to overcome key limitations of free drugs, such as poor aqueous solubility, rapid systemic clearance, nonspecific distribution, and dose-limiting toxicity [2,3,4]. By modulating particle size, surface charge, hydrophilicity, ligand density, and degradation behavior, nanomedicine platforms can enhance circulation time, promote preferential accumulation in diseased tissues, and provide controlled or stimuli-responsive release of therapeutic payloads [1,5]. These advantages have supported the development of various nanotherapeutic systems for cancer, inflammatory diseases, infectious diseases, neurological disorders, and cardiovascular diseases [6]. Despite these advances, conventional drug delivery-oriented nanomedicine has often been evaluated mainly by indirect therapeutic outcomes, such as changes in tumor volume, survival, histological response, or biomarker expression [7,8]. Although these endpoints are essential, they do not fully reveal how nanomedicines behave in vivo during the therapeutic process [9,10]. For example, a nanoparticle formulation may show accumulation in a target tissue but fail to release its payload efficiently, or it may release the therapeutic agent prematurely before reaching the intended site [11]. Similarly, therapeutic activation may occur only in a limited region of the disease microenvironment, while conventional endpoint analyses may not capture such spatial and temporal heterogeneity [12]. These limitations have created a growing need for nanomedicine systems that can provide real-time information on their biological fate and functional performance. Theranostic nanomedicine extends conventional nanomedicine by integrating therapeutic delivery with diagnostic imaging within a single nanoscale platform [13,14,15,16,17,18]. Conventional theranostic systems can visualize nanocarrier distribution, target-site accumulation, and treatment response, thereby supporting image-guided therapy. However, in many cases, the imaging signal primarily reflects the location of the nanocarrier or imaging probe and does not necessarily indicate whether the therapeutic payload has been released, the intended therapeutic mechanism has been activated, or a meaningful biological response has occurred [9,11,19,20]. This distinction establishes a clear conceptual hierarchy: conventional nanomedicine primarily focuses on improving drug delivery, conventional theranostic nanomedicine adds imaging-based localization and treatment guidance, and self-monitoring theranostic nanomedicine further provides functional feedback on events occurring after administration. Accordingly, increasing attention has been directed toward self-monitoring theranostic nanomedicines [21,22,23]. In this review, self-monitoring theranostic nanomedicines are defined as intelligent nanoscale platforms that generate measurable imaging or diagnostic signals reflecting their own biodistribution, payload release, therapeutic activation, and/or biological response in real time, near real time, or through longitudinal imaging [24]. Importantly, self-monitoring is considered here as a continuum of functional information rather than as a requirement for strictly continuous real-time signal acquisition. For conceptual clarity, we categorize self-monitoring into four functional levels: Level 1, biodistribution and biological fate monitoring; Level 2, payload release monitoring; Level 3, therapeutic activation monitoring; and Level 4, downstream biological response monitoring. These levels represent progressively more functionally informative stages of the therapeutic process and may be evaluated individually or in combination depending on the imaging modality and study design. Unlike conventional imaging-guided nanomedicines that mainly visualize where the nanocarrier accumulates, self-monitoring systems are designed to provide functional feedback on what happens after administration [25]. The concept of self-monitoring can be implemented through several design strategies [12,26,27]. Always-on imaging signals can be used to track systemic circulation, organ distribution, and target-site accumulation. Activatable probes can remain silent during circulation but generate signals in response to disease-associated cues such as acidic pH, reactive oxygen species, glutathione, enzymes, hypoxia, ATP, or specific biomarkers [28]. Förster resonance energy transfer-based systems, self-quenching/dequenching probes, ratiometric sensors, and environment-sensitive contrast agents can further enable visualization of payload release or intracellular activation [29,30,31,32]. In addition, multimodal imaging systems can combine the high sensitivity of optical or nuclear imaging with the superior spatial resolution or tissue penetration of magnetic resonance, computed tomography, ultrasound, or photoacoustic imaging [33,34]. Through these approaches, self-monitoring nanomedicines can provide more comprehensive information than conventional theranostic platforms [23]. Real-time or longitudinal monitoring of biodistribution, payload release, therapeutic activation, and biological response is particularly important for improving the reliability and translational potential of nanomedicine [9,18,23]. Biodistribution monitoring can clarify whether nanomedicines reach the target site while minimizing off-target accumulation in organs such as the liver, spleen, kidney, or lung [28]. Payload release monitoring can help distinguish true drug delivery from simple carrier accumulation and can identify premature release or insufficient release at the disease site [31,32]. Therapeutic activation monitoring is essential for treatment modalities such as prodrug therapy, photothermal therapy, photodynamic therapy, sonodynamic therapy, gene therapy, RNA therapy, and immunotherapy, where therapeutic efficacy depends on activation within a specific biological context. Finally, response monitoring can provide early feedback on apoptosis, metabolic changes, immune modulation, vascular remodeling, hypoxia alleviation, or other disease-associated biological outcomes before macroscopic changes become apparent [35,36]. Self-monitoring theranostic nanomedicines therefore represent an important step toward feedback-guided and precision nanomedicine. By generating dynamic information throughout the therapeutic process, these systems may allow optimization of dose, timing, treatment sequence, and patient-specific therapeutic strategies [23,32,35]. They may also help resolve a central challenge in nanomedicine research: the gap between nanoparticle design and actual in vivo performance. Instead of relying solely on final therapeutic outcomes, self-monitoring platforms can provide mechanistic insight into the relationship between nanoparticle delivery, payload release, therapeutic activation, and biological efficacy [9,35,36]. In this review, we discuss recent progress in self-monitoring theranostic nanomaterials for biomedical imaging and therapy, with particular emphasis on their applications in cancer theranostics. We first summarize the conceptual framework and design principles of self-monitoring nanomedicine, including key signal-generation mechanisms and biological triggers. We then discuss representative imaging strategies and nanoplatforms used for monitoring nanoparticle fate, payload release, therapeutic activation, and disease response. Cancer applications are highlighted to illustrate how tumor accumulation, intratumoral delivery, microenvironment-responsive activation, and therapeutic response can be visualized and integrated within a single nanotheranostic framework, while selected inflammatory and infectious disease applications demonstrate the broader biomedical relevance of these principles. Finally, we highlight current challenges related to quantitative signal reliability, biological safety, manufacturing reproducibility, regulatory complexity, and clinical translation, and we provide future perspectives on the development of clinically translatable self-monitoring theranostic nanomedicines.

## 2. Concept and Design Principles of Self-Monitoring Theranostic Nanomedicines

**Figure 1:**
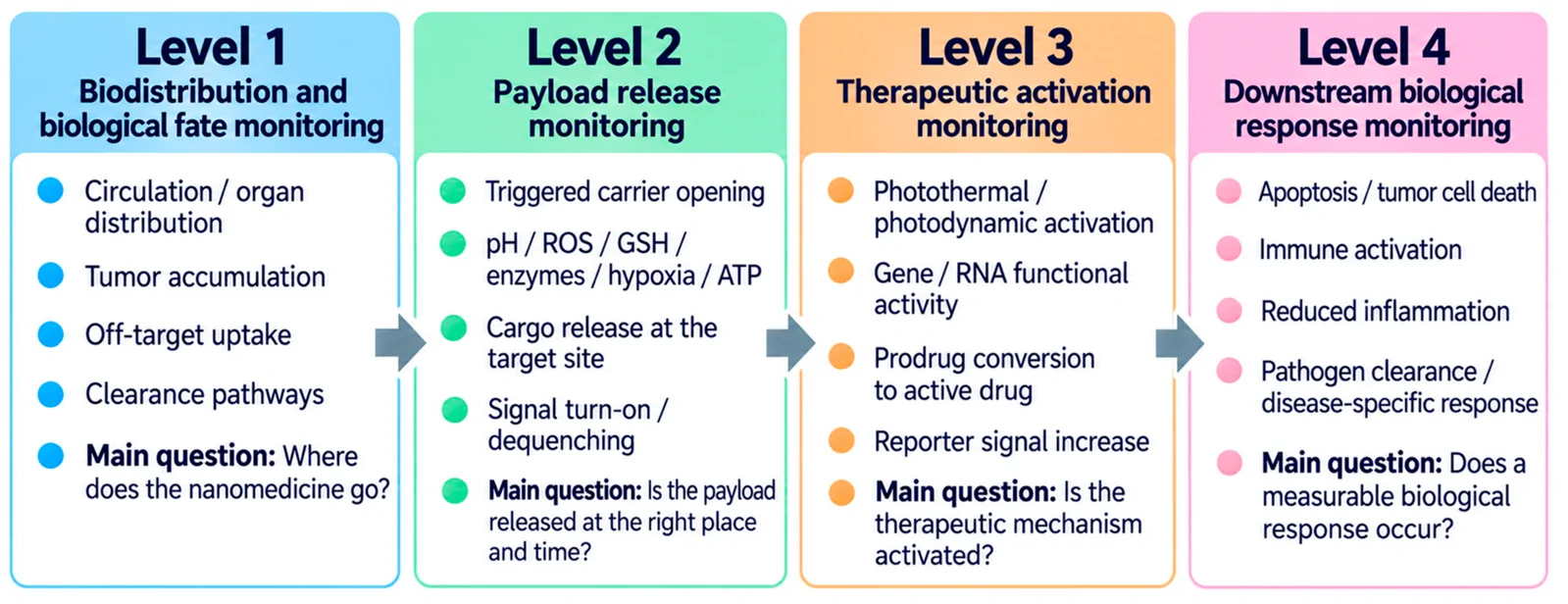
Conceptual framework of self-monitoring theranostic nanomedicines organized into four functional levels. Level 1 monitors nanomedicine biodistribution and biological fate, including circulation, target-site accumulation, off-target uptake, and clearance. Level 2 evaluates payload release in response to physiological or pathological triggers. Level 3 monitors activation of the therapeutic mechanism, including photothermal or photodynamic activation, gene/RNA activity, and prodrug conversion. Level 4 assesses downstream biological responses, such as apoptosis, immune activation, inflammation modulation, and pathogen clearance. These monitoring functions may be evaluated in real time, near real time, or through longitudinal imaging, depending on the imaging modality and study design.

Self-monitoring theranostic nanomedicines can be defined as multifunctional nanoscale systems that integrate therapeutic delivery, diagnostic imaging, and functional monitoring in real time, near real time, or through longitudinal imaging within a single platform. Unlike conventional nanomedicines that are primarily designed to improve pharmacokinetics, biodistribution, or target-site accumulation, self-monitoring theranostic systems are engineered to provide dynamic information on their own biological fate and therapeutic activity after administration [1,7,14,21,37]. These systems aim not only to visualize where the nanocarrier is located but also to determine whether the payload has been released, whether the therapeutic mechanism has been activated, and whether a measurable biological response has occurred [20,38]. This concept represents an important evolution from conventional theranostic nanomedicine. Traditional theranostic systems commonly combine imaging agents with therapeutic payloads to enable image-guided treatment; however, in many cases, the imaging signal primarily reflects the distribution of the nanocarrier or contrast agent rather than functional drug release or pharmacological activity [14,22]. For example, a fluorescent, magnetic, or radiolabeled nanoparticle may show strong accumulation at a tumor site, but the therapeutic payload may remain entrapped within the carrier, undergo premature leakage during circulation, or fail to reach the intracellular compartment required for therapeutic action [11,39]. Self-monitoring systems are therefore designed to reduce this uncertainty by linking imaging signals more directly to defined delivery, release, activation, or response events [32,40]. A self-monitoring theranostic nanomedicine generally consists of several key components: a nanocarrier matrix, a therapeutic payload, an imaging or monitoring probe, a stimuli-responsive linker or gatekeeper, and, when needed, a targeting or biomimetic surface. The nanocarrier matrix provides the structural framework for payload loading, protection, circulation, and controlled delivery, and may be composed of lipids, polymers, inorganic materials, organic–inorganic hybrids, biomimetic membranes, or extracellular vesicle-like systems [37,41]. The therapeutic payload may include small-molecule drugs, prodrugs, proteins, peptides, nucleic acids, photosensitizers, sonosensitizers, immune modulators, or gene-editing components [14,42]. Imaging or monitoring probes provide visual or quantitative readouts, while responsive linkers and gatekeepers enable stimulus-triggered signal activation or payload release. Targeting ligands and cell membrane coatings can further enhance site-specific accumulation, immune evasion, cellular uptake, or interaction with disease-specific microenvironments [41,43]. Among these components, the relationship between the imaging probe and the therapeutic payload is particularly important. In simple image-guided nanomedicine, the imaging moiety may be co-loaded or chemically attached to the nanocarrier without directly reporting payload release or therapeutic activation. In contrast, self-monitoring systems require a more integrated design in which the imaging signal changes in response to a specific biological or therapeutic event [9,44,45]. For example, the signal may be quenched while the payload remains encapsulated and restored after release, or the imaging readout may change according to linker cleavage, carrier degradation, intracellular trafficking, enzymatic activation, or cell death-associated biomarkers [46,47]. Therefore, the design of self-monitoring nanomedicines requires careful coordination among carrier stability, stimulus sensitivity, signal generation, and therapeutic function. Signal generation is a central design element of self-monitoring theranostic nanomedicine [48]. Always-on imaging signals can be used to monitor systemic circulation, organ distribution, target-site localization, and clearance, particularly when quantitative imaging modalities such as PET, SPECT, MRI, CT, or fluorescence imaging are employed [8,49,50]. However, always-on signals generally report the presence of the carrier or imaging probe rather than the occurrence of a functional therapeutic event. Thus, they are useful for monitoring biodistribution and biological fate but are less informative for assessing payload release or therapeutic activation. Activatable imaging signals provide a more functional approach. These systems remain silent or weakly detectable under normal physiological conditions but generate enhanced signals in response to disease-associated cues [40,51,52]. Activation can occur through bond cleavage, conformational change, aggregation or disaggregation, redox reaction, enzymatic degradation, or removal of a protective coating. This strategy can improve the signal-to-background ratio and increase specificity by reducing nonspecific signals during circulation while enhancing contrast at disease sites. Activatable systems are particularly useful for pathological microenvironments characterized by acidic pH, elevated reactive oxygen species, high intracellular glutathione, enzyme overexpression, hypoxia, ATP enrichment, or abnormal biomarker expression [51,53,54]. Ratiometric and Förster resonance energy transfer-based systems provide additional advantages for quantitative self-monitoring. In ratiometric systems, two signal channels are used, and the ratio between them reflects a biological or physicochemical event, reducing artifacts caused by probe concentration, imaging depth, excitation intensity, or tissue heterogeneity [55,56]. FRET-based systems are especially useful for monitoring payload release, nanocarrier disassembly, or intracellular trafficking because energy transfer efficiency depends strongly on the distance between donor and acceptor fluorophores [29,57]. When the nanocarrier remains intact, donor and acceptor molecules remain in close proximity and generate a characteristic signal. After carrier degradation or payload release, the separation of donor and acceptor changes the fluorescence readout, allowing release events to be visualized. Similar principles are used in self-quenching and dequenching systems, where fluorescence is suppressed at high local fluorophore concentration and restored after payload release or carrier disassembly [58,59]. Multimodal imaging is another important strategy for self-monitoring nanomedicine. Because no single imaging modality provides optimal sensitivity, spatial resolution, tissue penetration, temporal resolution, and quantitative accuracy, combining two or more modalities can provide complementary information [60,61]. Optical imaging offers high sensitivity and molecular responsiveness but limited tissue penetration. MRI provides excellent anatomical resolution and soft-tissue contrast but relatively low sensitivity. PET and SPECT provide sensitive and quantitative whole-body tracking but have limited spatial resolution and require radiolabeling. CT and ultrasound offer clinically established imaging workflows, while photoacoustic imaging combines optical contrast with ultrasound-based tissue penetration. By integrating these modalities, self-monitoring systems can more comprehensively evaluate biodistribution, payload release, therapeutic activation, and biological response [62,63]. Biological triggers are essential for the design of self-monitoring theranostic nanomedicines. Disease tissues often exhibit abnormal microenvironmental features that can be exploited for both therapeutic delivery and signal activation. Acidic pH can trigger protonation, charge conversion, carrier swelling, or acid-labile bond cleavage. Elevated ROS can activate oxidation-sensitive linkers or probes, while high intracellular glutathione can cleave disulfide-containing systems and promote cytosolic payload release [64,65]. Enzymes such as matrix metalloproteinases, cathepsins, hyaluronidase, esterases, and phospholipases can degrade specific peptide, polymeric, or lipid-based linkers. Hypoxia can activate reduction-sensitive probes or prodrugs, while ATP can trigger release from aptamer-, host–guest-, or mesoporous silica-based systems. Disease-associated receptors, metabolites, nucleic acids, and immune-related biomarkers can also be used to generate more specific monitoring responses [19,66,67,68]. For self-monitoring systems to be effective, the selected trigger should be matched to the biological question being addressed. For example, pH-responsive systems may be suitable for monitoring endosomal escape or acidic tumor microenvironments but may not directly indicate therapeutic response. ROS-responsive systems may reflect oxidative stress or photodynamic/sonodynamic activation, but background ROS in inflammatory tissues can complicate interpretation [69,70]. Enzyme-responsive systems can provide high biological specificity when the target enzyme is strongly associated with the disease state, but enzyme expression may vary by patient, disease stage, and tissue region [71,72]. Therefore, the trigger should not merely activate the nanomedicine; it should also provide interpretable information related to delivery, release, activation, or response. A major design requirement for self-monitoring theranostic nanomedicine is signal specificity. The monitoring signal should ideally correspond to a defined biological or therapeutic event rather than nonspecific carrier accumulation. For example, a signal intended to represent payload release should be minimally affected by nanocarrier concentration, tissue autofluorescence, serum protein adsorption, or premature probe leakage. Similarly, a signal intended to indicate therapeutic response should be distinguishable from signals generated by passive accumulation or carrier degradation. Achieving this level of specificity often requires activatable or ratiometric designs, appropriate control groups, and validation using complementary analytical methods [73,74]. Quantitative reliability is another critical consideration. Although many theranostic nanomedicines generate visually strong imaging signals, the relationship between signal intensity and biological function is not always rigorously established. For self-monitoring nanomedicines, it is important to determine whether the imaging signal quantitatively correlates with delivered dose, released payload amount, intracellular activation, or therapeutic efficacy. This requires calibration under physiologically relevant conditions, validation in complex biological media, and comparison with independent measurements such as drug quantification, histological analysis, molecular assays, or pharmacodynamic biomarkers [75,76]. Biocompatibility and safety must also be considered from the earliest stages of design. Self-monitoring theranostic nanomedicines are often more complex than conventional drug delivery systems because they combine carriers, therapeutic agents, imaging probes, responsive components, and targeting elements [77]. Each component may influence toxicity, immune recognition, biodistribution, degradation, and clearance. Inorganic imaging agents, persistent fluorophores, radiolabels, and heavy-metal-based contrast materials may raise concerns regarding long-term retention or organ toxicity. Surface modifications used to extend circulation or improve targeting may alter protein corona formation, macrophage uptake, and immune response [78,79]. Therefore, the additional monitoring function should not compromise the safety, therapeutic benefit, or translational feasibility of the nanomedicine. Finally, clinical translatability should guide the rational design of self-monitoring nanomedicine platforms. Although highly complex multifunctional systems may provide elegant proof-of-concept results, excessive structural complexity can hinder reproducibility, scale-up, quality control, regulatory evaluation, and clinical adoption [73,74,75,76]. Clinically viable self-monitoring nanomedicines should balance functionality with simplicity. Preferable designs may include biodegradable carriers, clinically acceptable imaging agents, scalable preparation methods, reproducible physicochemical properties, and compatibility with existing imaging workflows [80,81,82,83]. The ultimate goal is not merely to create nanomedicines with multiple functions, but to develop systems that provide actionable information capable of improving treatment decisions. Taken together, self-monitoring theranostic nanomedicines are designed through the integration of nanocarrier engineering, responsive chemistry, imaging science, and therapeutic function. Their central purpose is to transform nanomedicine from a passive delivery vehicle into an informative therapeutic platform that can reveal its own behavior and biological effects in real time. The four-level conceptual framework of self-monitoring theranostic nanomedicine is summarized in Figure 1 and provides the basis for the following sections, which discuss imaging strategies and representative approaches for monitoring biodistribution, payload release, therapeutic activation, and therapeutic response.

## 3. Imaging Strategies for Real-Time Monitoring

Imaging strategy is a central element in the design of self-monitoring theranostic nanomedicines because these systems require not only therapeutic delivery but also real-time visualization of nanomedicine behavior and therapeutic function in living systems [14,84,85]. Unlike conventional endpoint analysis, imaging can provide spatial and temporal information on systemic biodistribution, target-site accumulation, payload release, therapeutic activation, and early biological response. Therefore, imaging modalities should be selected according to the specific monitoring purpose. Fluorescence imaging is useful for cellular-level and activatable signal monitoring, MRI provides anatomical and soft-tissue contrast, PET and SPECT enable sensitive whole-body tracking, CT offers high-resolution anatomical information, ultrasound provides real-time and clinically accessible imaging, and photoacoustic imaging bridges optical contrast with deeper tissue visualization [10,18]. Because each imaging modality has distinct strengths and limitations in sensitivity, tissue penetration, spatial resolution, temporal resolution, quantification, and clinical compatibility, no single technique can fully capture all stages of self-monitoring theranostic nanomedicine performance [86]. For example, fluorescence imaging can sensitively report molecular activation or payload release but is limited in deep-tissue quantification, whereas PET or SPECT can provide quantitative whole-body biodistribution but may not directly distinguish intact nanocarriers from released or metabolized imaging components. Multimodal imaging strategies can help overcome these limitations by combining complementary readouts for localization, pharmacokinetic tracking, functional activation, and response evaluation [49]. In practical terms, the choice of a multimodal imaging combination should be guided by the biological question and disease-related monitoring requirement rather than by simply increasing the number of imaging modalities. For deep-seated or disseminated disease, PET/SPECT combined with CT or MRI can integrate quantitative whole-body tracking with precise anatomical localization. For lesions requiring real-time local intervention, fluorescence or photoacoustic imaging combined with ultrasound can provide sensitive functional information together with rapid spatial guidance. When the primary goal is to monitor payload release or therapeutic activation, activatable fluorescence/FRET or photoacoustic readouts can be paired with MRI or PET to distinguish local functional events from overall nanomedicine distribution. Thus, multimodal imaging is most useful when the selected modalities provide complementary, rather than redundant, information matched to the specific monitoring demand [49,60,61]. In this context, imaging is not merely a diagnostic component but a functional feedback tool that links nanomedicine design to in vivo performance. To provide a more practical comparison beyond modality-level characteristics, Table 1 summarizes representative self-monitoring platforms according to carrier or nanoplatform, monitoring role, trigger or signal mechanism, experimental setting, key advantage, main limitation, and reference. The following sections further discuss how each modality can visualize specific stages of nanomedicine delivery, activation, and therapeutic response.

### 3.1. Optical and Near-Infrared Fluorescence Imaging

Optical and near-infrared fluorescence imaging is one of the most powerful modalities for self-monitoring theranostic nanomedicine because fluorescence signals can be engineered to respond directly to nanoscale and molecular events. Unlike anatomical imaging modalities that primarily visualize tissue structure or contrast-agent distribution, fluorescence imaging can report changes in fluorophore intensity, emission wavelength, fluorescence lifetime, self-quenching/dequenching, or energy-transfer efficiency. These signal changes can be linked to nanoparticle circulation, target-site accumulation, intracellular trafficking, payload release, therapeutic activation, or biological response. Therefore, optical fluorescence imaging is particularly useful for mechanistic evaluation of self-monitoring nanomedicines, especially at cellular, tissue, and small-animal levels. Near-infrared fluorescence imaging has been especially important for in vivo nanomedicine monitoring because longer wavelengths reduce tissue autofluorescence and photon scattering compared with visible-light imaging. Early NIR-II imaging studies demonstrated that this spectral region could provide real-time visualization of deep-tissue biological processes. In one representative study, single-walled carbon nanotube fluorophores emitting in the second near-infrared window were used for high-frame-rate anatomical imaging in mice after intravenous injection. This study visualized dynamic nanoprobe circulation through major organs, including the lungs, kidneys, spleen, and liver, and showed that NIR-II imaging could provide improved anatomical resolution compared with conventional NIR-I imaging [87]. A subsequent vascular imaging study further demonstrated real-time NIR-II imaging using single-walled carbon nanotubes, achieving approximately 30 μm spatial resolution and less than 200 ms temporal resolution at tissue depths of 1–3 mm. Importantly, this platform allowed quantification of blood velocity in normal and ischemic femoral arteries, illustrating how NIR-II fluorescence can monitor dynamic biological function rather than static nanoparticle localization alone [96]. The development of more biocompatible and excretable NIR-II fluorophores has further advanced the translational potential of fluorescence-guided nanomedicine. The small-molecule NIR-II dye CH1055 was reported to show rapid renal excretion, with approximately 90% excreted through the kidneys within 24 h. This probe enabled high-resolution lymphatic imaging, noninvasive brain tumor imaging at approximately 4 mm depth, EGFR-targeted tumor imaging after affibody conjugation, and image-guided tumor resection [97]. These findings are important for self-monitoring nanomedicine because they show that NIR-II fluorophores can be designed not only for high-contrast imaging but also for improved biological clearance. In another study, rare-earth-doped nanoparticles with NIR-I and NIR-II emissive bands were developed, and NIR-II fluorescence imaging allowed more accurate detection of small and early tumor deposits than visible, NIR-I, luciferase, or red fluorescent protein-based imaging in an ovarian cancer dissemination model [98]. These studies indicate that NIR-II imaging can improve the sensitivity and spatial accuracy of disease-site monitoring. NIR-II fluorescence imaging has also been applied to image-guided surgery and tumor-margin delineation. An in vivo-assembled NIR-II downconversion nanoprobe system modified with DNA and targeting peptides was reported for metastatic ovarian cancer surgery. The assembled nanoprobes showed improved photostability, deep tissue penetration of approximately 8 mm, enhanced tumor-to-normal tissue contrast, and stable tumor retention for surgical guidance. Notably, ovarian metastases smaller than or equal to 1 mm could be completely excised under NIR-II fluorescence guidance [99]. This example is directly relevant to self-monitoring theranostics because it illustrates how disease-site nanoprobe assembly and optical readout can define the timing and location of intervention. Beyond intensity-based imaging, a lifetime-engineered NIR-II nanoparticle platform was developed for multiplexed in vivo imaging. By tuning luminescence lifetimes rather than relying only on intensity or emission wavelength, this platform resolved selected lifetime-encoded probes at tissue depths up to 8 mm, where intensity-based signal-to-noise ratios became poor [100]. Such lifetime-based strategies may improve quantitative reliability in complex tissue environments. For self-monitoring theranostic nanomedicine, the most important advantage of fluorescence imaging is that it can be coupled to payload release or therapeutic activation. Fluorescence signals can be designed to remain quenched during circulation and turn on after linker cleavage, carrier disassembly, redox activation, enzyme activation, or intracellular release. A reduction-responsive BODIPY–camptothecin conjugate was reported in which a disulfide linker connected the fluorophore and the drug. Upon reduction-triggered cleavage, free camptothecin was released and fluorescence emission increased, allowing self-reporting of drug release at the cellular level. The hydrophobic dye–drug conjugate also self-assembled into nanoparticles, supporting tumor delivery and in vivo therapy [101]. This type of design directly connects a therapeutic release event with a measurable optical signal. Ratiometric fluorescence designs can further improve signal reliability because they reduce dependence on probe concentration, excitation intensity, optical attenuation, and imaging conditions. ROS-activated ratiometric fluorescent polymeric nanoparticles were developed using PEG-containing amphiphilic block copolymers bearing oxidation-responsive phenylboronic ester motifs and a ROS-activatable fluorescent monomer. These nanoparticles could encapsulate doxorubicin, and H_2_O_2_-triggered doxorubicin release was correlated with changes in ratiometric fluorescence [102]. This study is a useful example of how a pathological stimulus, payload release, and optical readout can be integrated into one self-reporting nanomedicine platform. A photosensitizer-doped FRET nanoparticle system was also designed for self-monitoring and self-delivery in combination cancer therapy. In this system, fluorescence changes were used to monitor nanoparticle delivery and therapeutic processes, while photosensitizer loading supported photodynamic treatment [22]. These primary studies show that fluorescence imaging can serve several self-monitoring roles. First, always-on NIR or NIR-II fluorescence can track circulation, biodistribution, tumor accumulation, vascular dynamics, or surgical margins. Second, activatable fluorescence can report disease-associated stimuli such as redox state, ROS, pH, enzymes, or intracellular release. Third, FRET, self-quenching/dequenching, ratiometric fluorescence, and lifetime-encoded fluorescence can provide more mechanistically informative readouts than simple intensity imaging. These properties make optical and NIR fluorescence imaging particularly suitable for early-stage development of self-monitoring nanomedicines, where cellular localization, release behavior, and therapeutic activation must be evaluated in detail. Nevertheless, optical fluorescence imaging has important limitations. Even in the NIR-II region, signal intensity can still be influenced by tissue depth, absorption, scattering, probe concentration, detector sensitivity, and heterogeneous biodistribution. A strong tumor fluorescence signal may represent nanoparticle accumulation, fluorophore retention, payload release, therapeutic activation, or tissue clearance delay, depending on probe design. Therefore, fluorescence imaging should not be interpreted as direct evidence of therapeutic efficacy unless the signal mechanism is clearly defined and quantitatively validated. Future fluorescence-based self-monitoring nanomedicines should prioritize ratiometric or lifetime-based readouts, calibration under biologically relevant conditions, correlation with actual payload release or therapeutic biomarkers, and integration with clinically compatible modalities when deeper or more quantitative whole-body monitoring is required.

### 3.2. Magnetic Resonance Imaging

**Figure 2:**
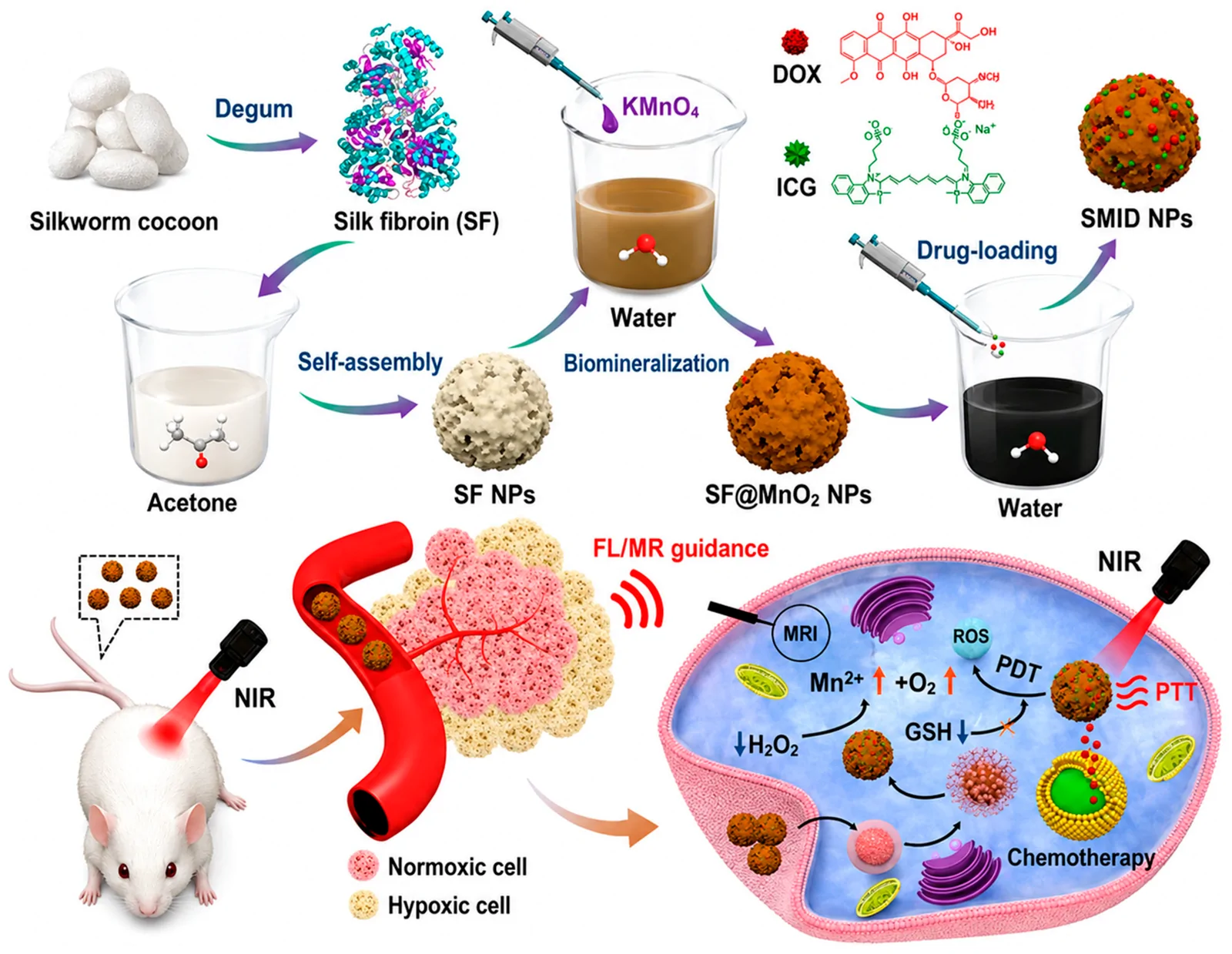
Preparation and tumor-microenvironment-responsive theranostic functions of SMID nanoparticles for MR/fluorescence imaging-guided chemotherapy, photothermal therapy, and photodynamic therapy. Adapted from Ref. [105] under the terms of the Creative Commons Attribution 4.0 International License (CC BY 4.0).

Magnetic resonance imaging is a clinically established modality that provides excellent anatomical resolution, soft-tissue contrast, and deep-tissue visualization without ionizing radiation. In self-monitoring theranostic nanomedicine, MRI is particularly useful for tracking nanomedicine accumulation in deep tissues, evaluating anatomical localization, and detecting microenvironment-responsive contrast changes [88,103,104,105,106]. MRI-based nanomedicines commonly use superparamagnetic iron oxide nanoparticles, manganese oxide nanostructures, gadolinium-containing materials, or other paramagnetic components to generate T1-weighted, T2-weighted, or dual-mode contrast depending on material composition, size, magnetic properties, water accessibility, and degradation behavior. MRI has been used not only for anatomical localization but also for monitoring functional nanomedicine delivery. A magnetic nanoparticle-based siRNA delivery system enabled tumor accumulation to be visualized by MRI and near-infrared fluorescence imaging, while gene silencing was confirmed using optical reporter imaging [103]. This study showed that MRI can be integrated with therapeutic delivery and functional response evaluation. Similarly, magnetism-engineered iron oxide nanoprobes conjugated with Herceptin enabled sensitive in vivo molecular MRI of cancer marker expression, demonstrating the value of magnetic nanoparticles for target-specific tumor imaging [104]. Manganese-based nanoplatforms are especially useful for self-monitoring MRI because Mn^2+^ can produce strong T1-weighted contrast after microenvironment-triggered degradation or ion release. Tumor-microenvironment-responsive MnO_2_ systems provide representative examples of this strategy. In a hollow mesoporous MnO_2_ nanoplatform co-loaded with doxorubicin and chlorin e6, acidic tumor conditions induced MnO_2_ decomposition, drug release, oxygen generation, and Mn^2+^-mediated T1-weighted MRI enhancement [88]. In this system, longitudinal relaxivity increased markedly from 0.051 mM^−1^ s^−1^ at pH 7.4 to 8.743 mM^−1^ s^−1^ after incubation at pH 6.0 for 6 h, and T1-weighted MRI showed approximately twofold tumor signal enhancement 24 h after intravenous injection in 4T1 tumor-bearing mice [88]. This example is particularly suitable for illustrating self-monitoring MRI because contrast generation is mechanistically connected to tumor-responsive degradation, payload release, oxygen generation, and therapeutic activation. MRI can also be combined with other imaging and therapeutic modalities. A silk fibroin-templated MnO_2_ nanocomplex co-loaded with indocyanine green and doxorubicin enabled MR/fluorescence imaging-assisted trimodal cancer therapy, where MnO_2_ reacted with tumor H_2_O_2_ to generate oxygen and enhance photodynamic therapy, as illustrated in Figure 2 [105]. A redox-responsive hybrid nanogel system containing MnO_2_ nanoparticles and doxorubicin showed glutathione-triggered Mn^2+^ release for enhanced T1 MRI and doxorubicin release for chemotherapy; ultrasound-targeted microbubble destruction further improved tumor penetration and therapeutic efficacy [106]. These examples show how MRI can be integrated with fluorescence, ultrasound, chemotherapy, photodynamic therapy, and microenvironment modulation.

Despite these advantages, MRI has several limitations for self-monitoring theranostic nanomedicine. MRI is less sensitive than fluorescence or nuclear imaging, and detectable contrast changes generally require sufficient accumulation of contrast-generating materials. In addition, MRI signal intensity can be influenced by water accessibility, local tissue properties, magnetic susceptibility, aggregation state, metal ion release, degradation kinetics, and clearance. Therefore, MRI signal enhancement should not be directly interpreted as payload release or therapeutic response unless it is validated by drug release assays, histology, pharmacodynamic markers, or complementary imaging. Overall, MRI is powerful for deep-tissue and anatomical monitoring, but its self-monitoring value depends on clear mechanistic coupling between contrast change, nanomaterial transformation, payload release, and therapeutic function.

### 3.3. Photoacoustic Imaging

Photoacoustic imaging is a hybrid imaging modality that combines optical excitation with ultrasound detection. When light-absorbing molecules or nanoparticles absorb pulsed laser energy, transient thermoelastic expansion generates acoustic waves that can be detected by ultrasound transducers. Because acoustic waves scatter less than photons in biological tissues, photoacoustic imaging provides deeper tissue visualization than conventional optical imaging while retaining strong optical absorption-based contrast. In self-monitoring theranostic nanomedicine, this modality is particularly useful for tracking nanomaterial accumulation, monitoring vascular distribution, evaluating oxygenation or hypoxia, and guiding light-mediated therapies such as photothermal or photodynamic therapy [32,89,107,108,109]. Several research studies have demonstrated the usefulness of photoacoustic imaging for functional nanomedicine monitoring. Semiconducting polymer nanoparticles were reported as photoacoustic molecular imaging probes with narrow spectral profiles, high photostability, and resistance to oxidation. Importantly, this platform enabled ratiometric photoacoustic imaging of reactive oxygen species in living mice, showing that photoacoustic signals can report dynamic pathological processes rather than simple nanoparticle localization [89]. In another semiconducting polymer nanoparticle system, intraparticle molecular orbital engineering enhanced photoacoustic brightness and photothermal therapy efficacy. The engineered nanoparticles showed 2.6-fold higher photoacoustic signal and 1.3-fold higher maximum photothermal temperature compared with control nanoparticles, allowing improved tumor photoacoustic imaging and photothermal ablation in vivo [107]. Stimuli-responsive photoacoustic probes are especially relevant to self-monitoring theranostic nanomedicine because their signals can be directly linked to microenvironmental activation or payload release. A photothermal stimuli-responsive composite nanomodulator based on PNIPAM-coated nanoparticles was developed for dynamic contrast-enhanced photoacoustic imaging. Repeated external laser stimulation produced reversible photoacoustic signal modulation, and background subtraction improved imaging contrast by approximately 6.9-fold ex vivo and 5.3-fold in vivo [108]. This strategy is useful because dynamic signal modulation can help distinguish responsive nanoprobes from endogenous absorbers such as blood. In another study, a perylene diimide-based nanotheranostic platform enabled ratiometric photoacoustic imaging of tumor acidic pH and simultaneous monitoring of pH-responsive drug release in living mice [32]. This example directly connects tumor microenvironment sensing, drug release, and photoacoustic readout. Photoacoustic imaging can also guide therapeutic activation, especially in photothermal nanomedicine. A reduced graphene oxide–gold nanoparticle sandwich nanostructure was developed for NIR-II photoacoustic imaging-guided photothermal therapy in ovarian cancer. The plasmonic coupling between gold nanoparticles and reduced graphene oxide generated strong NIR-II absorption, enhanced photoacoustic signals, and efficient photothermal conversion under 1061 nm laser irradiation. In tumor-bearing mice, photoacoustic imaging showed tumor accumulation with the strongest signal at 24 h post-injection, supporting image-guided photothermal treatment [109]. This type of platform demonstrates how photoacoustic imaging can be used to determine when sufficient nanomaterial has accumulated before applying external irradiation. Despite these advantages, photoacoustic imaging has several limitations. Signal intensity depends on optical absorption, laser wavelength, tissue depth, local light fluence, acoustic detection geometry, and the photophysical properties of the contrast agent. Therefore, increased photoacoustic signal should not automatically be interpreted as payload release, therapeutic activation, or biological response unless the signal mechanism is clearly validated. For self-monitoring theranostic nanomedicine, photoacoustic imaging is most valuable when contrast changes are mechanistically coupled to a defined event, such as nanoparticle accumulation, pH-responsive transformation, drug release, ROS generation, oxygenation change, or photothermal activation. Overall, photoacoustic imaging provides an important bridge between optical molecular sensitivity and ultrasound-based deep-tissue visualization, but quantitative interpretation requires careful calibration and complementary validation.

### 3.4. PET and SPECT Imaging

**Figure 3:**
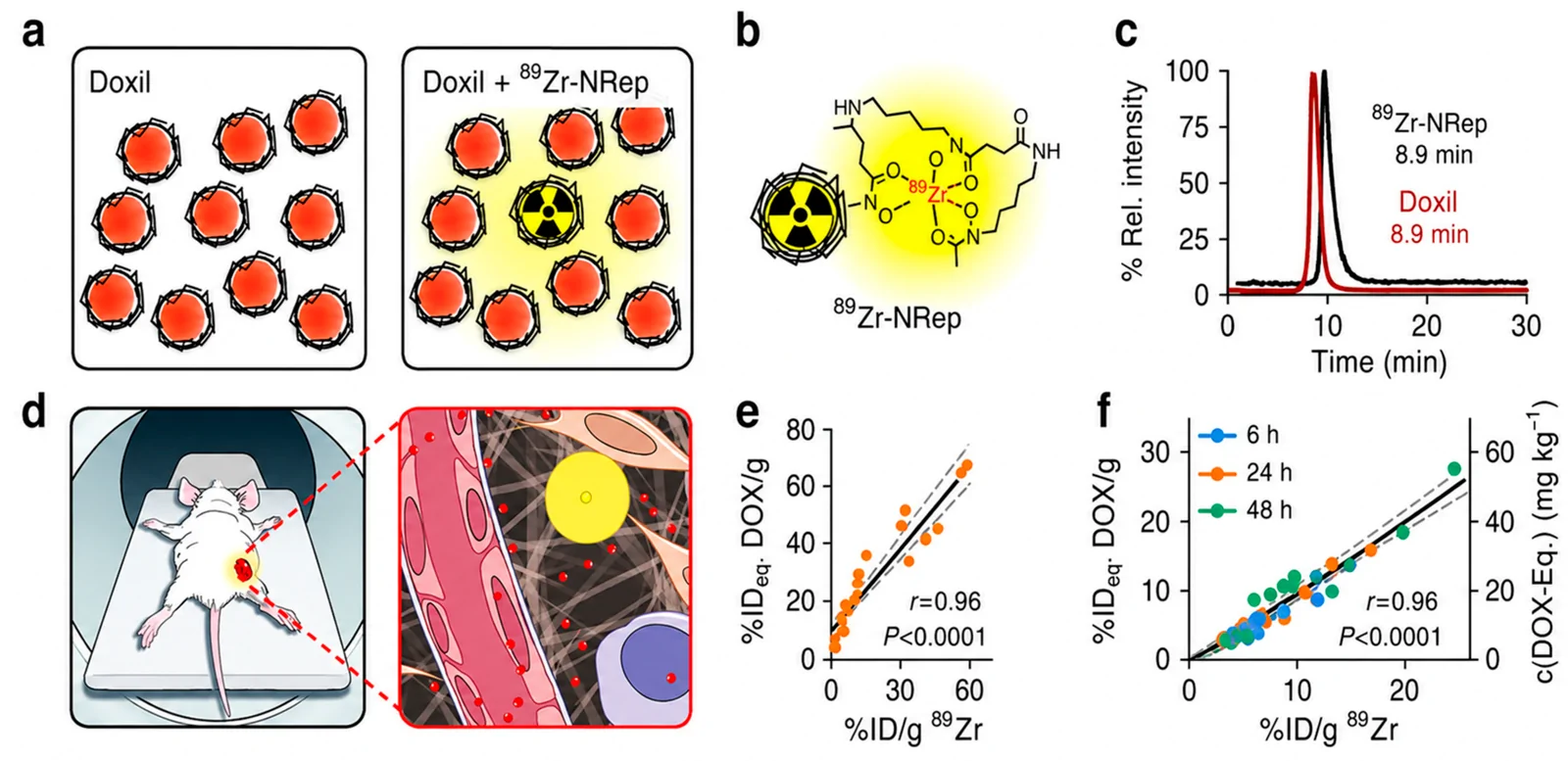
Companion PET nanoreporter for noninvasive quantification of liposomal doxorubicin delivery. (**a**,**b**) Schematic of Doxil co-administered with the DFO-chelated ^89^Zr-labeled liposomal nanoreporter (^89^Zr-NRep). (**c**) Comparable size-exclusion retention times of Doxil and ^89^Zr-NRep indicate similar physicochemical behavior. (**d**) PET imaging enables noninvasive assessment of nanoreporter accumulation and DOX delivery. (**e**,**f**) ^89^Zr-NRep uptake strongly correlated with DOX concentrations in blood and tumor tissues at 6, 24, and 48 h after administration. Thus, increased PET signal from ^89^Zr-NRep serves as a surrogate readout of greater liposomal DOX delivery to the tumor. Adapted from Ref. [110] under the terms of the Creative Commons Attribution 4.0 International License (CC BY 4.0).

Positron emission tomography and single-photon emission computed tomography are highly sensitive nuclear imaging modalities that enable quantitative whole-body tracking of radiolabeled nanomedicines. In self-monitoring theranostic nanomedicine, PET and SPECT are particularly useful for evaluating pharmacokinetics, systemic biodistribution, tumor or disease-site accumulation, organ clearance, and intersubject variability in delivery efficiency [90,91,110,111,112]. Because radiotracers can be detected at very low concentrations, nuclear imaging can provide quantitative information even when nanoparticle accumulation is insufficient for optical or MRI-based detection. A representative PET nanoreporter strategy showed how nuclear imaging can predict nanotherapy efficacy. In this approach, a Zirconium-89-labeled liposomal nanoreporter was co-injected with Doxil without chemically modifying the clinical drug formulation [110]. The nanoreporter had similar physicochemical properties to Doxil and enabled noninvasive PET-based estimation of doxorubicin tumor delivery. Importantly, PET-derived nanoreporter uptake strongly correlated with doxorubicin concentration in tumors, and tumor uptake heterogeneity was observed independently of tumor size. Mice with more than 25 mg kg^−1^ doxorubicin accumulation in tumors showed significantly improved tumor growth inhibition and survival, demonstrating that PET can be used not only for biodistribution imaging but also for feedback-guided prediction of therapeutic outcome. The companion nanoreporter design and the correlation between ^89^Zr-NRep uptake and DOX delivery are illustrated in Figure 3.

Clinical translation of PET-based nanoparticle imaging has also been demonstrated using ultra-small hybrid optical-PET silica nanoparticles. In a first-in-human study, iodine-124-labeled integrin-targeting silica nanoparticles were evaluated in patients with metastatic melanoma. The platform combined PET-based whole-body pharmacokinetic assessment with optical imaging potential and showed renal clearance, tumor targeting, and an acceptable safety profile [90]. This study is important because it demonstrates that carefully designed radiolabeled nanoparticles can move beyond preclinical imaging and provide clinically relevant information on biodistribution, clearance, and target engagement. SPECT imaging has similarly been used for clinical nanotheranostic monitoring. A phase 0 exploratory study evaluated Rhenium-188-labeled PEGylated liposomes in patients with metastatic tumors using serial whole-body imaging and SPECT/CT at 1, 4, 8, 24, 48, and 72 h after injection. This study provided pharmacokinetic, biodistribution, dosimetry, tumor targeting, and safety information in patients. Blood and plasma terminal half-lives were 36.73 ± 14.00 h and 52.02 ± 45.21 h, respectively, and organ dosimetry values were below reference tolerance limits [91]. This example illustrates the value of SPECT/CT for patient-level evaluation of nano-radiotherapeutic biodistribution and dosimetry. Radiolabeling design is critical for accurate PET and SPECT interpretation. A generic Zirconium-89 labeling method for liposomal nanoparticles achieved near-quantitative radiochemical yields of approximately 97–98% for different liposomal formulations and enabled PET-based analysis of heterogeneous tumor distribution, kidney accumulation, and differential metabolic fate of free versus liposome-encapsulated radiolabels [111]. Similarly, Gallium-68/Gallium-67-labeled sphingolipid nanoemulsions were developed for PET and SPECT imaging, showing that radionuclide choice and surface modification can be used to tune biodistribution and imaging time windows [112]. These studies emphasize that nuclear imaging signals must be interpreted in relation to radiolabel stability, nanoparticle integrity, and clearance pathways. Despite their strengths, PET and SPECT also have important limitations in self-monitoring theranostic nanomedicine. Nuclear imaging usually reports the distribution of the radiolabel, which may not always represent the intact nanocarrier, released payload, or therapeutic activation state. Radiolabel detachment, nanoparticle degradation, or differential clearance of labeled components can produce misleading biodistribution signals. In addition, PET and SPECT require specialized radiochemistry, imaging infrastructure, radiation safety procedures, and regulatory consideration. Therefore, PET/SPECT-based self-monitoring should include careful radiolabel stability testing, ex vivo biodistribution validation, and, when possible, correlation with drug concentration, therapeutic activation markers, or biological response. Overall, nuclear imaging is highly valuable for quantitative whole-body monitoring, but its mechanistic interpretation depends on whether the radiolabel faithfully reports the intended biological event.

### 3.5. Computed Tomography and Ultrasound Imaging

Computed tomography is widely used in clinical practice because of its high spatial resolution, rapid acquisition, and compatibility with anatomical imaging workflows. In nanomedicine, CT contrast is commonly generated using high atomic number elements such as gold, bismuth, tantalum, iodine, hafnium, or other radiopaque materials. Although CT is generally less sensitive than fluorescence or nuclear imaging, CT-compatible nanoparticles can provide useful information on anatomical localization, tumor accumulation, vascular distribution, and image-guided therapy [92,113,114]. Several research studies have demonstrated the potential of CT-compatible nanoparticles for theranostic monitoring. A bismuth sulfide–mesoporous silica nanoplatform loaded with doxorubicin was developed for CT imaging and NIR-responsive osteosarcoma therapy. After RGD peptide modification, the nanoparticles showed selective osteosarcoma accumulation, with approximately tenfold higher accumulation in tumor cells than in peritumoral tissues, and the Bi_2_S_3_ core enabled CT imaging while also supporting photothermal therapy and NIR-triggered drug release [92]. A more recent gold nanodot system conjugated with 2-deoxy-D-glucose was designed as a tumor-targeted CT contrast agent and radiosensitizer. This platform used tumor glucose uptake as a targeting mechanism and combined CT visualization with enhanced radiotherapeutic efficacy [113]. In another CT-focused study, tantalum oxide nanoparticles with 9–12 nm diameters and high tantalum content were developed as versatile CT contrast agents. These nanoparticles enabled micro-CT visualization of full-body vasculature for approximately 3 h after intravenous injection and were also formulated into mesoporous silica or PLGA carriers as CT-imagable drug delivery vehicles [114]. These examples show that CT can support self-monitoring mainly by visualizing high-Z nanomaterial localization and anatomical distribution, although CT signal alone usually does not directly indicate payload release or therapeutic response. Ultrasound imaging is another clinically established modality with real-time imaging capability, low cost, portability, and no ionizing radiation. In theranostic nanomedicine, ultrasound-responsive microbubbles, nanobubbles, phase-change nanodroplets, and gas-generating nanoparticles can be used for imaging, drug delivery, and externally triggered therapy [93,115,116]. Unlike CT, ultrasound can also provide mechanical energy for therapeutic activation. Acoustic cavitation, acoustic droplet vaporization, sonoporation, microbubble destruction, and increased vascular permeability can enhance local payload release and tissue penetration. Phase-change nanodroplets provide representative examples of ultrasound-based self-monitoring and triggered release. A programmable low-intensity focused ultrasound-triggered nanodroplet system encapsulated both perfluoropentane and doxorubicin. Under focused ultrasound exposure, the nanodroplets converted into microbubbles, generated ultrasound contrast, and released doxorubicin after microbubble collapse [93]. Two types of droplets with different shell stiffness required different ultrasound parameters for activation; lipid nanodroplets were activated at 3 W for 3 min, whereas PLGA nanodroplets required 8 W for 3 min. This design enabled sequential and programmable drug release, including an additional approximately 20% drug release after the second ultrasound trigger [93]. This study is directly relevant to self-monitoring nanomedicine because the ultrasound signal was associated with phase transition and externally controlled drug release. More recent systems have integrated ultrasound with photoacoustic imaging to monitor externally triggered release. In an optical-responsive phase-change nanodroplet platform, ultrasound and photoacoustic imaging were used to monitor NIR-triggered optical droplet vaporization and drug release in tissue [115]. This approach allowed imaging signal changes to be used for determining the optimal activation time point after nanodroplet accumulation. Ultrasound-targeted microbubble destruction has also been applied to modulate the tumor microenvironment and enhance immunotherapy. In a 4T1 breast cancer model, low-intensity focused ultrasound-targeted microbubble destruction reduced tumor blood perfusion, induced immunogenic cell death features such as calreticulin exposure, increased dendritic cell and CD8^+^ T-cell responses, and sensitized tumors to anti-PD-L1 therapy [116]. These examples show that ultrasound can be used not only for imaging and delivery but also for monitoring and modulating therapeutic activation and biological response. Despite these advantages, both CT and ultrasound have limitations for self-monitoring theranostic nanomedicine. CT requires relatively high local concentrations of radiopaque materials and involves ionizing radiation, while long-term retention of inorganic high-Z nanoparticles may raise safety concerns. Ultrasound-based monitoring can be affected by contrast-agent stability, circulation time, acoustic sensitivity, imaging window, and spatial heterogeneity of ultrasound exposure. Microbubbles are generally confined to the vascular space, whereas nanobubbles and phase-change droplets require careful optimization to balance circulation, tissue penetration, acoustic activation, and therapeutic payload release. Therefore, CT and ultrasound signals should be interpreted carefully and, when possible, correlated with drug release, histology, pharmacodynamic markers, or complementary imaging. Overall, CT is most useful for anatomical localization of high-Z nanomedicines, whereas ultrasound is particularly valuable for real-time, externally controlled, and feedback-guided nanomedicine activation.

### 3.6. Complementary Imaging Modalities

Several complementary imaging modalities can further support self-monitoring nanomedicine. Magnetic particle imaging (MPI) is particularly useful for quantitative monitoring because it directly detects magnetic nanoparticle tracers with negligible tissue background. Notably, an MPI nanocomposite showed a strong linear correlation between MPI signal changes and doxorubicin release (R^2^ = 0.99) and enabled quantitative drug-release monitoring in tumor-bearing mice [11]. Bioluminescence/BRET and chemiluminescence can provide highly sensitive optical signals without external light excitation. A dye-loaded nanobubble system used MPO-dependent luminol emission followed by BRET/FRET to enhance inflammation-associated optical signals in vivo [94], whereas chemiluminescence-based probes are useful for detecting disease-associated biochemical events with low background signals [72]. Raman spectroscopy, particularly surface-enhanced Raman scattering (SERS), provides molecularly specific spectral information and can be used to monitor payload release. For example, a pH-responsive SERS nanoplatform enabled real-time monitoring of doxorubicin release through recovery of the Raman reporter signal under acidic conditions [95]. Together, these complementary modalities extend self-monitoring beyond simple nanocarrier localization by providing quantitative, biochemical, or molecular-level information.

## 4. Monitoring Biodistribution and Payload Release

The therapeutic performance of nanomedicines is strongly governed by their in vivo fate after systemic or local administration. Even when a nanomedicine is rationally engineered with optimized size, surface chemistry, targeting ligands, and stimuli-responsive properties, its biological activity ultimately depends on whether it can circulate for a sufficient period, avoid rapid clearance, reach the intended tissue, penetrate biological barriers, release the payload at the appropriate site, and interact with target cells [1,7]. These processes are highly dynamic and cannot be fully predicted from formulation properties alone. For example, experimental analysis of hard nanomaterial clearance showed that liver sequestration is strongly influenced by blood-flow dynamics, organ microarchitecture, and interactions with specific hepatic cell populations [117]. In another study, a companion magnetic resonance imaging nanoparticle was able to quantify heterogeneous tumor accumulation and predict the response of therapeutic nanoparticles, demonstrating that delivery efficiency itself can be a determinant of therapeutic outcome [118]. Therefore, real-time monitoring of biodistribution and payload release is essential for linking nanomedicine design to actual in vivo performance. Conventional nanomedicine studies often evaluate biodistribution and payload release using ex vivo organ fluorescence, tissue extraction, histology, blood sampling, or endpoint pharmacokinetic analysis [9]. Although these methods provide important quantitative and mechanistic information, they generally offer limited temporal resolution and may not capture dynamic changes in circulation, organ accumulation, carrier degradation, tissue penetration, or payload release. This distinction is particularly important because target-site accumulation does not necessarily indicate functional delivery. A nanocarrier may accumulate in diseased tissue but fail to release its payload, release it prematurely during circulation, or deliver it to non-target cells or inappropriate intracellular compartments. Self-monitoring theranostic nanomedicines are designed to address these limitations by generating imaging signals that report nanoparticle fate, carrier integrity, payload release, or microenvironment-triggered activation in living systems [9,31]. For instance, an ATP-responsive mesoporous-silica-coated multicolor upconversion nanoparticle system enabled long-term tracking and real-time monitoring of intracellular drug release through luminescence signal changes [31]. Such approaches can help distinguish simple carrier localization from functional payload availability and provide mechanistic insight into why a formulation succeeds or fails in vivo.

### 4.1. Biodistribution and Biological Fate Monitoring

Biodistribution monitoring is one of the most fundamental functions of self-monitoring theranostic nanomedicine. After administration, nanomedicines encounter multiple biological barriers, including serum proteins, immune cells, vascular endothelium, extracellular matrix, cellular membranes, and clearance organs. These interactions determine circulation time, organ distribution, target-site accumulation, tissue penetration, cellular uptake, and eventual elimination [43,119,120,121]. Because these processes are strongly affected by nanoparticle size, shape, surface chemistry, charge, stiffness, and biological environment, real-time monitoring is essential for determining whether the actual in vivo behavior of a nanomedicine matches its intended design [43,119,120,121]. Blood circulation and protein adsorption are early determinants of nanomedicine biodistribution. Once nanoparticles enter biological fluids, proteins and other biomolecules can rapidly adsorb onto their surface and form a biomolecular corona, which may alter the biological identity of the nanomedicine and influence immune recognition, macrophage uptake, complement activation, and systemic clearance [43]. Particle size, surface charge, hydrophilicity, shape, rigidity, and surface coating further determine circulation half-life and organ distribution [119]. Renal clearance studies using quantum dots showed that urinary elimination of inorganic nanoparticles strongly depends on hydrodynamic size and surface chemistry, demonstrating that small differences in particle design can markedly change clearance pathways [122]. Therefore, self-monitoring systems that incorporate fluorescence, PET, SPECT, MRI, or photoacoustic signals can provide time-dependent information on circulation kinetics and systemic availability. Systemic biodistribution is also shaped by interactions with clearance organs. The liver and spleen are major sites of nanoparticle sequestration because of sinusoidal filtration, macrophage uptake, and mononuclear phagocyte system activity, whereas the kidney contributes to elimination of small nanoparticles, degradation products, and molecular imaging probes [43,119,120,122]. Monitoring organ distribution is therefore important not only for evaluating delivery efficiency but also for assessing potential toxicity, long-term retention, and clearance behavior. For self-monitoring nanomedicines, a persistent signal in an organ should be interpreted carefully because it may represent intact carrier accumulation, released payload, degraded imaging components, or clearance of dissociated probes. Target-site accumulation is a major goal of nanomedicine design, particularly in cancer and inflammatory diseases. In solid tumors, nanoparticle accumulation has traditionally been explained by the enhanced permeability and retention effect, but delivery efficiency varies widely depending on tumor type, vascular density, stromal composition, interstitial pressure, and disease stage [120]. Active targeting strategies may improve cellular recognition and retention, but they do not necessarily guarantee deep tissue penetration or improved therapeutic efficacy [120]. Recent tumor transport studies further showed that nanoparticle entry into solid tumors cannot be explained only by passive leakage through inter-endothelial gaps; active trans-endothelial transport can also contribute substantially to tumor entry [123]. This finding emphasizes why real-time and multiscale monitoring is needed to understand how nanomedicines actually reach diseased tissues. A critical issue in biodistribution monitoring is that target-site accumulation should not be interpreted as equivalent to therapeutic delivery. Imaging signals associated with a nanocarrier can confirm localization but may not indicate whether the payload has been released, whether the carrier has penetrated beyond perivascular regions, or whether the payload has reached the appropriate intracellular compartment. For example, a multistage nanoparticle delivery system was designed to combine long circulation with improved tumor penetration, showing that both systemic availability and intratumoral diffusion are necessary for effective delivery into dense tumor tissue [124]. Such studies highlight the importance of monitoring not only whole-body biodistribution but also tissue-level distribution, intratumoral penetration, cellular uptake, and subcellular trafficking. Biological barrier transport is also important in non-tumor applications. For neurological delivery, the blood–brain barrier restricts the entry of most therapeutic agents and nanocarriers through tight junctions, efflux transporters, pericytes, astrocytic end-feet, and basement membrane structures [121]. Nanomedicine strategies for brain delivery often rely on receptor-mediated transport, surface engineering, focused ultrasound-mediated barrier opening, or biomimetic approaches [121]. Dense polyethylene glycol coatings have been shown to improve the penetration of polymeric nanoparticles within brain tissue, indicating that surface engineering can strongly affect movement through complex extracellular environments [125]. Imaging-based self-monitoring is particularly valuable in this context because brain delivery efficiency is often low and difficult to quantify using endpoint analysis alone. Overall, biodistribution and biological fate monitoring provide essential information for evaluating nanomedicine performance beyond conventional endpoint efficacy. By tracking circulation, organ distribution, target-site accumulation, clearance, and barrier transport, self-monitoring theranostic nanomedicines can reveal whether a formulation behaves as intended in vivo. More importantly, these systems can help identify the specific biological step at which delivery fails, such as rapid clearance, off-target sequestration, poor tissue penetration, insufficient cellular uptake, or ineffective intracellular trafficking. Therefore, biodistribution monitoring should ideally be designed as a multiscale process that connects whole-body pharmacokinetics with tissue-level localization, cellular delivery, and functional payload availability.

### 4.2. Payload Release Monitoring

**Figure 4:**
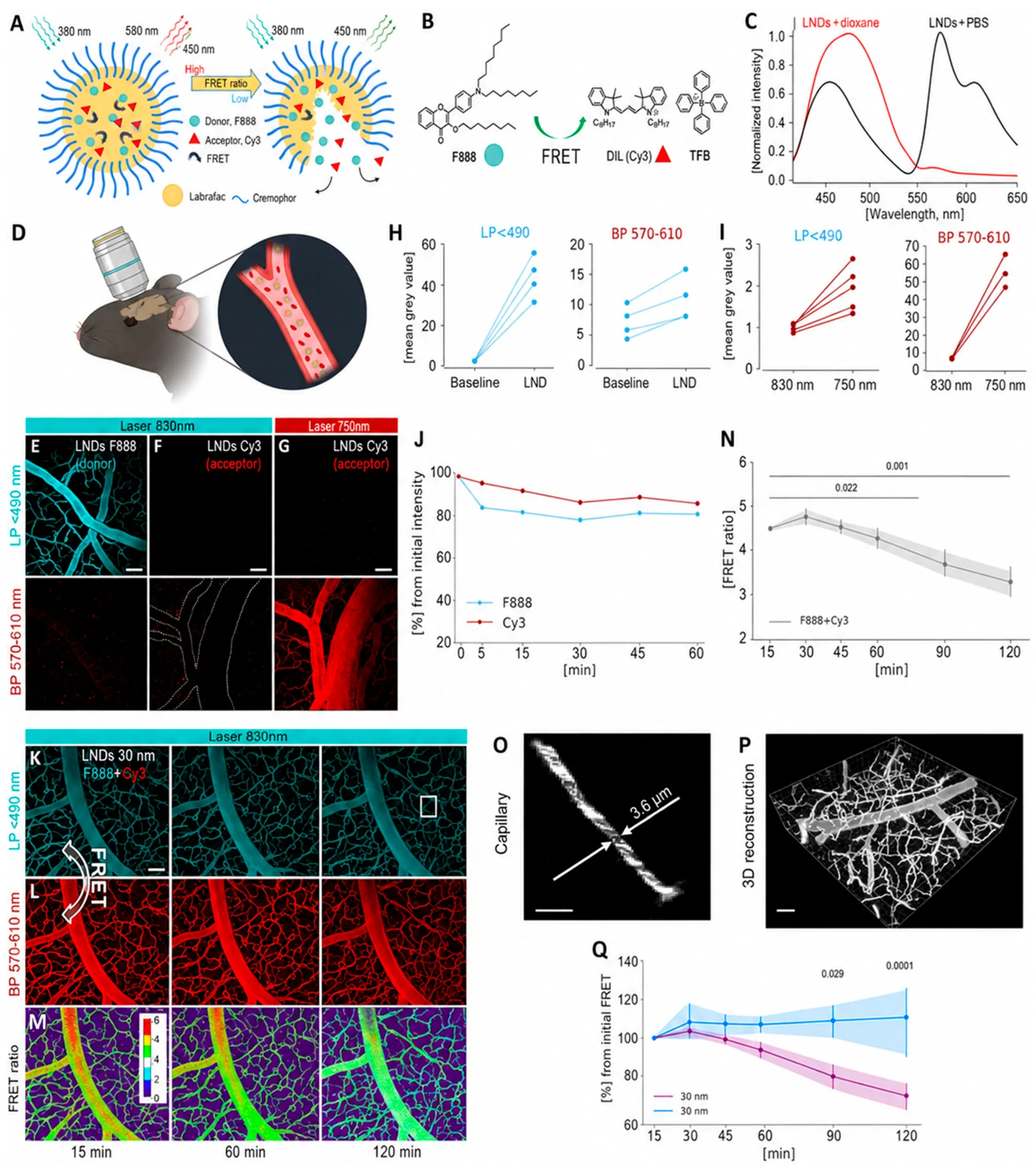
Real-time intravital monitoring of lipid nanodroplet disintegration using Förster resonance energy transfer (FRET). (**A**,**B**) Design and molecular components of FRET-enabled lipid nanodroplets containing the donor F888 and acceptor DiI–tetraphenylborate. Nanodroplet disintegration increases donor–acceptor separation and decreases the FRET ratio. (**C**) Fluorescence spectra of the nanodroplets in intact and disrupted states. (**D**–**M**) Cranial-window two-photon imaging and ratiometric visualization of circulating nanodroplets in mouse cortical vasculature. The white squares in (**K**) indicate the regions of interest (ROIs) selected for magnified observation. (**N**) Time-dependent reduction in the FRET ratio, indicating progressive nanodroplet disintegration in vivo. (**O**,**P**) High-resolution capillary imaging and three-dimensional reconstruction of the brain vasculature. (**Q**) Comparison of the in vivo degradation profiles of 30 and 80 nm nanodroplets. Accordingly, a progressive decrease in the FRET ratio provides a direct optical readout of nanodroplet disintegration and loss of structural integrity in vivo. Adapted from Ref. [130] under the terms of the Creative Commons Attribution 4.0 International License (CC BY 4.0).

Although biodistribution monitoring provides essential information on where nanomedicines travel and accumulate, it does not necessarily indicate whether therapeutic payloads are released at the desired site. This distinction is important because nanocarriers may accumulate in diseased tissues but still fail therapeutically if the payload remains trapped, is released too slowly, leaks prematurely during circulation, or does not reach the appropriate intracellular compartment [19,126]. Therefore, payload release monitoring is a key function of self-monitoring theranostic nanomedicine and provides functional information beyond carrier localization. Conventional release evaluation is commonly based on in vitro release assays, tissue extraction, blood sampling, or endpoint pharmacokinetic analysis. Although these methods are useful for formulation screening, they may not accurately reflect the dynamic in vivo environment, where protein adsorption, enzymatic activity, pH gradients, redox conditions, interstitial pressure, cellular uptake, and intracellular trafficking can strongly affect release behavior [19,126]. Self-monitoring release systems are designed to address this limitation by generating imaging signals that change in response to carrier disassembly, linker cleavage, payload diffusion, or microenvironment-triggered activation [28,126]. FRET-based systems are among the most widely used strategies for monitoring nanocarrier integrity and payload release. In these systems, donor and acceptor fluorophores are positioned close enough to allow energy transfer when the carrier remains intact; after release, degradation, or separation of labeled components, the FRET signal decreases or the emission ratio changes [28]. A representative mesoporous silica nanoparticle system used coumarin-labeled cysteine and FITC-β-cyclodextrin as a redox-responsive molecular valve, allowing real-time visualization of pore opening and drug release through FRET changes [29]. This type of design is useful because it can distinguish intact nanocarriers from dissociated or released components more effectively than simple fluorescence labeling. Fluorescence turn-on systems provide another practical approach for release monitoring. A dual turn-on controlled release system was constructed by conjugating doxorubicin and a fluorescent dye through an acid-cleavable hydrazone linker. Under acidic conditions, linker hydrolysis released both doxorubicin and the fluorescent dye, producing two fluorescence signals that enabled real-time monitoring of drug release dynamics in living cells and tumor tissues [127]. This example shows how release chemistry, imaging signal generation, and therapeutic payload activation can be directly integrated into one molecular design. Multimodal release-monitoring systems can provide more comprehensive information on payload availability in vivo. A core–satellite nanomedicine composed of ICG/DOX-loaded gelatin nanoparticles surrounded by CuS nanoparticles enabled enzyme-activatable release monitoring by fluorescence and photoacoustic dual-modal imaging. In this system, ICG fluorescence was initially quenched by CuS satellites, and fluorescence recovery was proportional to the amount of released doxorubicin after enzyme-triggered nanomedicine degradation [128]. A pH-responsive CuS-encapsulated ZIF-8 metal–organic framework system similarly used fluorescence recovery of doxorubicin to monitor drug release after acidic degradation of the carrier, while CuS supported photothermal therapy [129]. These examples demonstrate that payload release can be monitored through signal changes associated with carrier degradation, FRET disruption, or recovery of quenched fluorescence. Recent studies also show that release monitoring can be extended to complex biological barriers and more quantitative imaging readouts. A real-time FRET-based lipid nanodroplet system was used to monitor nanocarrier drug release and blood–brain barrier penetration at post-stroke microthrombi, demonstrating that FRET imaging can provide dynamic information on both vascular localization and cargo release in a neurologically relevant disease model**,** as illustrated in Figure 4 [130]. In another approach, a magnetic particle imaging nanocomposite showed a linear correlation between MPI signal changes and doxorubicin release over time, with an R^2^ value of 0.99 in vitro, and enabled quantitative drug release monitoring in tumor-bearing mice [11]. These studies are important because they move beyond qualitative “signal-on” release imaging toward more spatially and quantitatively interpretable release monitoring.

Despite these advances, payload release monitoring remains challenging. Imaging signal activation does not always directly correspond to the amount of released therapeutic payload, intracellular bioavailability, or pharmacological effect. Fluorescence recovery can be affected by probe concentration, tissue absorption, local pH, enzymatic activity, carrier degradation, and clearance of released components. Similarly, MRI, photoacoustic, or MPI signal changes may reflect material transformation or probe separation rather than therapeutic payload action itself. Therefore, self-monitoring release systems should be validated using complementary methods, including chemical drug quantification, histology, pharmacodynamic biomarkers, intracellular localization analysis, and therapeutic efficacy assessment. Taken together, payload release monitoring provides a crucial bridge between biodistribution imaging and therapeutic response assessment. Within the four-level framework introduced above, biodistribution and biological fate monitoring (Level 1) determines whether a nanomedicine reaches the intended tissue, whereas payload release monitoring (Level 2) establishes whether the therapeutic cargo becomes available at the appropriate time and location. These stages precede therapeutic activation (Level 3), which determines whether the intended treatment mechanism is initiated, followed by downstream biological response monitoring (Level 4), which evaluates whether activation produces a measurable therapeutic effect. Thus, biodistribution, release, activation, and response should be interpreted as sequential but distinct biological events, because success at one stage does not necessarily guarantee success at the next. FRET, fluorescence dequenching, dual turn-on probes, photoacoustic readouts, and quantitative magnetic imaging provide useful strategies for visualizing these transitions, but their ultimate value depends on the mechanistic relationship between imaging signal, payload availability, therapeutic activation, and biological efficacy.

## 5. Monitoring Therapeutic Activation and Response

Consistent with this sequential framework, successful biodistribution and payload release do not necessarily guarantee therapeutic efficacy. After a nanomedicine reaches the target tissue and releases its payload, the therapeutic mechanism must still be activated within the appropriate biological context. This distinction is particularly important for self-monitoring theranostic nanomedicines because many advanced therapeutic systems are not constitutively active but depend on chemical, physical, intracellular, or immunological activation. Prodrug-based systems require enzymatic or chemical conversion into active drugs, photodynamic therapy requires light irradiation and oxygen-dependent reactive oxygen species generation, RNA therapeutics require endosomal escape and functional engagement with intracellular machinery, and immunotherapeutic nanomedicines must activate immune pathways or remodel the immune microenvironment [131,132,133]. Therapeutic activation monitoring therefore represents a critical intermediate step between payload release and therapeutic response. Payload release monitoring can indicate that a therapeutic agent has become available, but it does not prove that the intended treatment mechanism has been initiated. This problem is evident in nucleic acid delivery, where cellular uptake alone does not ensure functional gene regulation. In one live-cell imaging study, lipid-formulated siRNA release from maturing endosomes was visualized within approximately 5–15 min after endocytosis and correlated with target gene knockdown, showing that endosomal escape and downstream gene silencing must be evaluated separately from cellular uptake [134]. This type of study highlights why self-monitoring systems should be designed to report functional activation, not merely carrier localization or payload presence. Therapeutic response monitoring is the next stage, determining whether activation produces a measurable biological effect. Response signals may include apoptosis, necrosis, metabolic suppression, vascular disruption, immune activation, or microenvironmental remodeling. A reporter nanoparticle platform demonstrated that anticancer efficacy could be monitored in real time by detecting treatment-induced apoptotic response in tumor models, providing an early readout of therapeutic activity before conventional endpoint changes become evident [35]. Similarly, granzyme B-targeted PET imaging was used to detect cytotoxic immune activity and predict response to cancer immunotherapy, illustrating how imaging can move beyond drug delivery to assess whether the intended biological response has occurred [135]. Therefore, in self-monitoring theranostic nanomedicine, activation and response monitoring should be considered distinct but connected layers of feedback that link payload release to therapeutic outcome. Together, therapeutic activation and response monitoring provide mechanistic information that cannot be obtained from biodistribution or release imaging alone. Activation monitoring asks whether the therapeutic process has started, whereas response monitoring asks whether the target tissue has changed as a consequence of treatment. This distinction can help optimize treatment timing, external stimulus parameters, dosing schedules, and combination therapy strategies. It can also help identify failure mechanisms, such as inadequate intracellular activation, insufficient photothermal or photodynamic effect, poor endosomal escape, weak immune activation, or biological resistance despite apparently successful delivery. Thus, this section focuses on how self-monitoring nanomedicines can visualize therapeutic activation and early biological response as functional indicators of treatment performance.

### 5.1. Therapeutic Activation Monitoring

Therapeutic activation monitoring refers to the real-time visualization or indirect assessment of whether a delivered therapeutic system has entered an active functional state. In self-monitoring theranostic nanomedicine, activation may involve chemical conversion, physical energy transduction, intracellular release, gene regulation, immune stimulation, or microenvironmental modulation [136,137,138]. Unlike conventional imaging-guided delivery, which mainly confirms nanocarrier localization, activation monitoring aims to determine whether the therapeutic mechanism has actually been initiated at the target site. This distinction is essential because a nanomedicine may accumulate efficiently and even release its payload, yet still fail therapeutically if the activation condition is insufficient, spatially heterogeneous, or biologically mismatched. Chemotherapy and prodrug-based nanomedicines provide a representative example of why activation monitoring is needed. Many prodrug systems are designed to remain inactive or less toxic during circulation and become activated only after enzymatic cleavage, redox reaction, pH-triggered bond hydrolysis, hypoxia-associated reduction, or light-mediated transformation [136,137]. When the chemical activation process is coupled to a fluorescence, photoacoustic, MRI, or ratiometric signal change, the imaging readout can help distinguish simple payload release from pharmacological activation. This is particularly important because released therapeutics may still be ineffective if they remain trapped in endosomes, are exported by efflux pumps, or fail to reach the appropriate intracellular compartment. Recent responsive therapeutic systems further illustrate how microenvironmental and external stimuli can be coordinated with treatment activation. A pH/NIR dual-responsive implantable nanofiber patch incorporating CuS nanoparticles, polydopamine, and doxorubicin used acidic pH and NIR-II-induced heating to promote controllable drug release while combining photothermal therapy with chemotherapy [139]. Similarly, cyano-modulated Zn(II)-Schiff base complexes enabled lysosome-targeted fluorescence imaging and acidic-lysosome-responsive camptothecin release through pH-sensitive imine bonds, linking subcellular imaging with stimulus-responsive chemotherapy [140]. Photothermal therapy is another modality in which activation monitoring is critical because therapeutic efficacy depends on sufficient nanomaterial accumulation, light delivery, photothermal conversion, and local temperature elevation. A photoacoustic thermometer system was used to continuously monitor both effective photothermal agent distribution and temperature in tumor-bearing mice using three-dimensional photoacoustic/ultrasound dual-modal imaging [141]. This study showed that the photoacoustic distribution of photothermal agents could be correlated with the effective treatment area, suggesting that photoacoustic monitoring may help determine the optimal administration-to-irradiation interval before photothermal therapy [141]. Such feedback is valuable because excessive heating can damage normal tissues, whereas insufficient heating can lead to incomplete tumor ablation. Photodynamic and sonodynamic therapies require activation of photosensitizers or sonosensitizers to generate cytotoxic reactive oxygen species. In an activatable photodynamic nanoplatform, ZnPc@TPCB nanoparticles were designed to self-report photosensitizer activation and therapeutic effect through dual fluorescence/photoacoustic imaging [24]. Nanoparticle disassembly released zinc phthalocyanine and turned on red fluorescence, allowing photosensitizer activation to be monitored before and during photodynamic therapy [24]. For sonodynamic therapy, red blood cell membrane-enveloped O_2_ self-supplementing biomimetic nanoparticles were developed to alleviate tumor hypoxia and enhance ultrasound-triggered reactive oxygen species generation [142]. In this system, fluorescence imaging guided nanoparticle localization, endogenous H_2_O_2_ was converted to O_2_ through the natural enzyme system of red blood cell membranes, and ultrasound activation generated reactive oxygen species for enhanced sonodynamic tumor therapy [142]. These examples show that activation monitoring can report not only sensitizer distribution but also whether light- or ultrasound-triggered therapeutic chemistry has been initiated. Gene and RNA therapy activation involves different monitoring challenges because therapeutic function requires intracellular bioavailability rather than simple cellular uptake. Nanomedicines delivering siRNA, mRNA, plasmid DNA, antisense oligonucleotides, or gene-editing components must overcome endosomal entrapment and release their payload into the cytosol before gene regulation or protein expression can occur. A Galectin-9 imaging assay was developed to quantify nanoparticle uptake, endosomal escape, and functional mRNA delivery in a single high-throughput platform [143]. This assay showed that Galectin-9 recruitment can serve as a quantitative reporter of endosomal escape events and can be correlated with mRNA translation [143]. This type of approach is highly relevant to self-monitoring nucleic acid nanomedicine because it separates three events that are often mistakenly conflated: nanoparticle uptake, endosomal escape, and functional gene expression. Beyond nucleic acid regulation, intracellular therapeutic activation can also involve targeted protein degradation. Antibody-enriched biomolecular condensates incorporating a proteasome-targeting motif enabled cytosolic delivery, direct proteasome recruitment, selective degradation of oncogenic KRAS G12V, and tumor suppression in a xenograft model [144]. Although this platform was not designed as an imaging-based self-monitoring system, it illustrates an emerging intracellular therapeutic endpoint that could be incorporated into future activation-monitoring theranostic strategies. Immunotherapeutic activation monitoring is also increasingly important because nano-immunotherapies depend on immune pathway activation rather than payload delivery alone. Nanomedicine platforms may deliver immune checkpoint inhibitors, STING agonists, Toll-like receptor agonists, cytokines, antigens, adjuvants, or mRNA vaccines, but their therapeutic outcome depends on dendritic cell maturation, antigen presentation, T-cell priming, macrophage repolarization, or remodeling of the immunosuppressive microenvironment. An imaging-guided self-amplifying photo-immunotherapeutic nanoparticle was recently designed to combine photoacoustic/ultrasound imaging, photothermal activation, and STING agonist delivery [145]. Under near-infrared irradiation, photothermal effects promoted phase transition and cGAMP release, which activated the STING pathway, enhanced dendritic cell maturation and cytotoxic T-cell activation, and generated antitumor immune responses against both primary and metastatic tumors [145]. This example illustrates how therapeutic activation monitoring can be linked to immune pathway initiation rather than only tumor accumulation. Despite these advances, therapeutic activation monitoring remains challenging. Activation signals must be distinguished from payload release signals and downstream response signals. For example, reactive oxygen species generation may indicate photodynamic or sonodynamic activation, but it may also reflect pre-existing oxidative stress or inflammation. Similarly, immune-associated imaging signals may indicate pathway activation but may not necessarily predict durable therapeutic benefit. Therefore, activation-monitoring systems should be designed so that the imaging signal is mechanistically coupled to a defined therapeutic event, such as prodrug conversion, heat generation, reactive oxygen species production, endosomal escape, gene expression, or immune pathway activation. Overall, therapeutic activation monitoring provides an essential bridge between payload release and therapeutic response, enabling self-monitoring nanomedicines to determine whether a delivered therapeutic system has entered its active state before downstream biological outcomes become apparent.

### 5.2. Therapeutic Response Monitoring

Therapeutic response monitoring represents the final and most biologically meaningful stage of self-monitoring theranostic nanomedicine. While biodistribution monitoring reveals where nanomedicines travel, payload release monitoring indicates whether therapeutic agents become available, and activation monitoring shows whether the therapeutic mechanism has been initiated, response monitoring determines whether the target tissue undergoes a measurable biological change. These responses may include apoptosis, necrosis, metabolic suppression, oxygenation change, vascular remodeling, immune activation, or microenvironmental reprogramming [146,147,148]. This distinction is important because successful accumulation, release, or activation does not always lead to therapeutic efficacy. Apoptosis and cell death imaging are among the most direct approaches for evaluating therapeutic response. Caspase-3 is widely used as an apoptosis-related imaging target because it is closely associated with execution-phase apoptosis and can be detected using substrate- or inhibitor-based probes [146]. In nanomedicine, this concept can be extended to therapeutic self-reporting systems. A programmed nanoparticle with self-adapting properties was designed to combine prolonged circulation, tumor targeting, receptor-mediated internalization, endo/lysosomal escape, controllable drug release, and in vivo fluorescence-based therapeutic self-reporting [149]. In this system, drug-induced apoptosis activated caspase-3, which generated a fluorescence signal, allowing therapeutic efficacy to be monitored in vivo and in real time [149]. This example shows how response monitoring can move beyond simple drug delivery and provide feedback on whether the delivered therapy actually induces tumor cell death. Recent self-reporting systems have further integrated response monitoring with treatment optimization. A real-time cell death self-reporting photodynamic theranostic agent was developed to visualize photosensitizer accumulation and report tumor cell death during photodynamic therapy. This system used cell-death-associated potassium ion efflux as a response indicator and enabled dynamic adjustment of therapeutic timing and irradiation parameters [25]. Such designs are valuable because they allow therapy to be optimized according to biological response rather than fixed treatment protocols. In self-monitoring nanomedicine, this type of response-sensitive readout can help distinguish insufficient activation from true therapeutic resistance. Tumor microenvironmental changes also provide important response indicators. Hypoxia and oxygenation are particularly relevant because they strongly influence photodynamic therapy, radiotherapy, chemotherapy, immunotherapy, and tumor progression [147]. Photoacoustic imaging has been used to evaluate oxygen-related tumor responses because it can visualize hemoglobin oxygenation and oxygen-sensitive contrast agents in living tissues [147]. In one research study, oxygen-sensitive tumor-targeted photoacoustic nanosonophores were used to map tumor oxygen distribution in patient-derived triple-negative breast cancer xenografts [150]. The initial oxygen distribution predicted spatial variation in radiotherapy efficacy, with poorly oxygenated regions showing lower treatment response [150]. This example illustrates how response monitoring can identify treatment-resistant microregions before anatomical changes become apparent. Immune response monitoring is another important component of therapeutic response assessment, especially for nano-immunotherapy and immunogenic cell death-inducing treatments. Granzyme B is a useful imaging target because it reflects cytotoxic immune cell activity and immune-mediated tumor killing [148]. In one PET imaging study, granzyme B-targeted PET enabled mapping of immune activation kinetics and magnitude across whole tumors after immune checkpoint therapy [151]. The imaging signal helped distinguish immunologically active tumors and provided mechanistic information that could be combined with flow cytometry, cytokine analysis, or sequencing-based validation [151]. More recently, a humanized melanoma mouse model was used to evaluate a ^68^Ga-labeled granzyme B PET tracer for early assessment of immunotherapy response. Tumor uptake of the tracer was higher in the combination immune checkpoint inhibitor group and correlated with tumor size reduction, indicating that immune response imaging can provide early information on therapeutic efficacy [152]. Despite these advances, therapeutic response monitoring remains challenging. Imaging signals must be interpreted carefully because cell death, inflammation, hypoxia, immune activation, and tissue remodeling can overlap temporally and spatially. For example, increased reactive oxygen species may indicate therapeutic activation, oxidative damage, or inflammation, depending on context. Similarly, immune-associated signals may reflect productive antitumor immunity, transient inflammation, or immune-related adverse events. Therefore, response-monitoring systems should be validated using independent biological measurements, such as caspase activity, TUNEL staining, cleaved PARP, histology, flow cytometry, cytokine profiling, immune cell phenotyping, oxygenation analysis, or tumor growth inhibition. Overall, therapeutic response monitoring provides a functional link between nanomedicine design and actual therapeutic benefit. It can reveal whether payload release and therapeutic activation lead to meaningful biological outcomes, such as tumor cell death, microenvironmental remodeling, or immune-mediated cytotoxicity. For self-monitoring theranostic nanomedicines, the most informative systems are those in which each imaging signal answers a distinct biological question: carrier localization, payload release, therapeutic activation, or biological response. By separating these layers of feedback, response-monitoring nanomedicines can support earlier treatment evaluation, optimization of therapeutic timing, identification of resistance mechanisms, and more adaptive treatment strategies.

## 6. Cancer Theranostics and Broader Biomedical Applications

Self-monitoring theranostic nanomedicines have broad potential across diverse biomedical fields, with cancer representing the most extensively investigated application because of the strong need to integrate precise tumor imaging with therapeutic delivery and response assessment. In contrast to conventional nanomedicine systems that mainly evaluate delivery efficiency or endpoint therapeutic outcomes, self-monitoring platforms can provide real-time or longitudinal information on disease-site accumulation, payload release, therapeutic activation, and biological response [80,153]. In cancer, these capabilities are particularly important for evaluating tumor accumulation, intratumoral penetration, microenvironment-responsive release, therapeutic activation, and early treatment response in heterogeneous tumor tissues [80,153]. The same self-monitoring principles can also be extended to inflammatory and immune-related diseases, neurological disorders, cardiovascular diseases, and infectious diseases [80,153,154,155,156]. In inflammatory and immune-related diseases, relevant monitoring targets include reactive oxygen species, inflammatory enzymes, macrophage activity, cytokine-related signaling, and vascular permeability [148,154]. For example, a serial ROS-responsive theranostic micelle system containing a two-photon aggregation-induced emission fluorophore and prednisolone was used for high-resolution inflammation imaging and anti-inflammatory therapy in acute lung injury, arthritis, and atherosclerosis models [157]. In neurological diseases, self-monitoring strategies should focus on blood–brain barrier transport, neuroinflammation, oxidative stress, and parenchymal delivery. In cardiovascular diseases, plaque inflammation, thrombosis, oxidative stress, endothelial dysfunction, and tissue perfusion are major monitoring targets [155]. In infectious diseases, pathogen-associated enzymes, acidic or hypoxic infection microenvironments, biofilm matrix barriers, and antimicrobial response are particularly important [156]. Thus, although cancer theranostics provides the principal framework for integrating nanomaterial-based imaging with therapeutic monitoring, disease-specific adaptations demonstrate the broader applicability of self-monitoring nanomedicine. The monitoring signal should be matched to the most relevant disease process: nanoparticle penetration in solid tumors, inflammatory activation in immune-mediated diseases, blood–brain barrier crossing in neurological disorders, plaque or thrombus activity in cardiovascular disease, and biofilm penetration or pathogen-responsive activation in infection. Such disease-specific monitoring can help distinguish passive accumulation from functional delivery, reveal whether therapeutic activation occurs in the correct biological context, and provide early evidence of treatment response before conventional endpoints become apparent.

### 6.1. Cancer Theranostics

**Figure 5:**
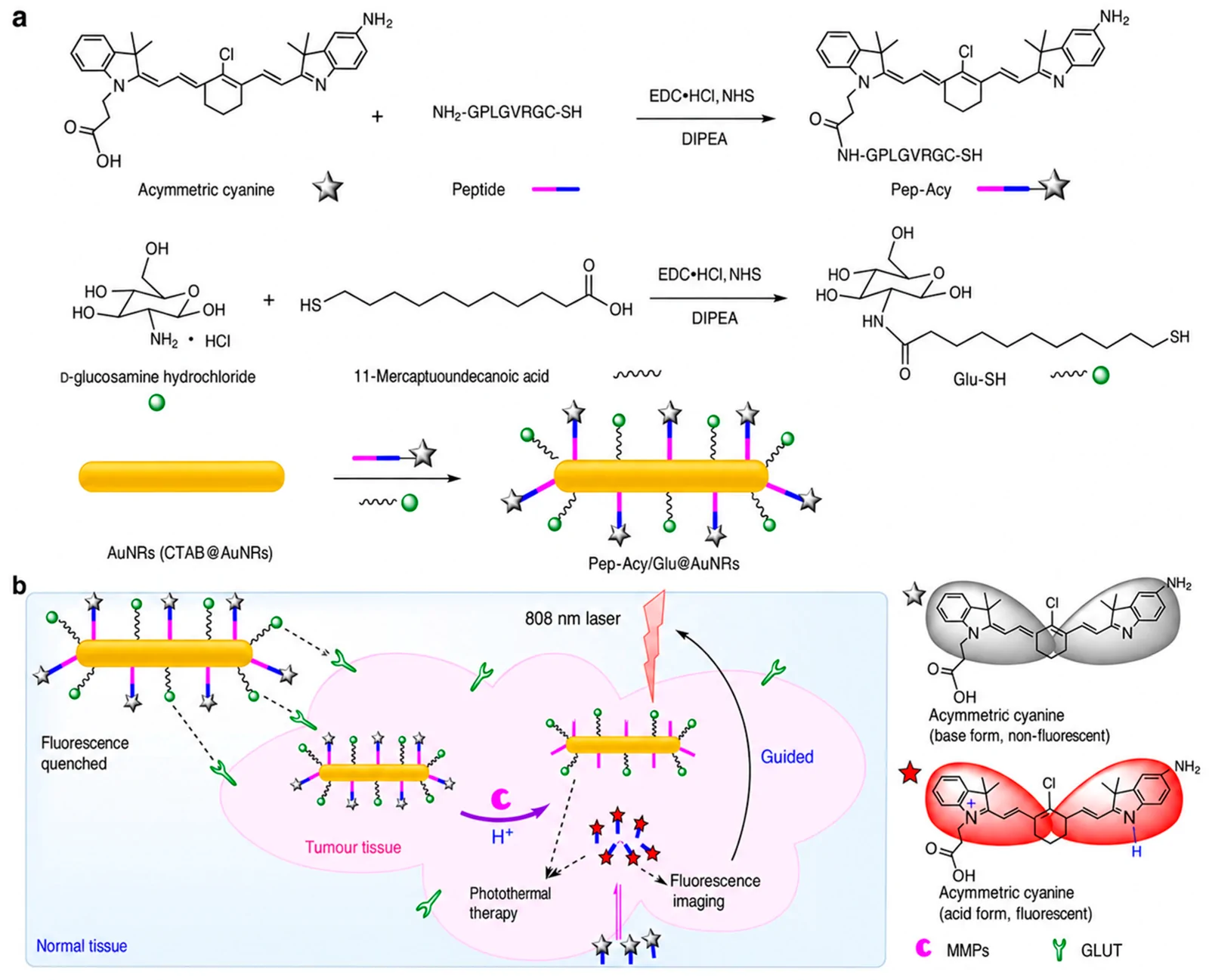
Dual-stimuli-responsive gold nanorod theranostic platform for tumor-specific fluorescence imaging and photothermal therapy. (**a**) Synthesis of asymmetric cyanine–peptide and glucosamine conjugates and their surface functionalization onto gold nanorods. (**b**) Glucose transporter-mediated tumor targeting and matrix metalloproteinase-responsive peptide cleavage facilitate nanoprobe accumulation and activation, while acidic tumor conditions reversibly convert the asymmetric cyanine from a quenched base form to a fluorescent acid form. The activated near-infrared fluorescence enables tumor localization and guides 808 nm laser-mediated photothermal therapy. Thus, fluorescence turn-on reflects tumor-microenvironment-responsive nanoprobe activation and provides a functional signal for determining the site and timing of photothermal treatment. Adapted from Ref. [161] under the terms of the Creative Commons Attribution 4.0 International License (CC BY 4.0).

Cancer is the most extensively investigated application area for self-monitoring theranostic nanomedicine because tumors exhibit multiple pathological features that can be exploited for imaging-guided delivery, stimuli-responsive activation, and response monitoring. These features include leaky vasculature, impaired lymphatic drainage, acidic extracellular pH, elevated glutathione levels, increased reactive oxygen species, hypoxia, abnormal enzyme expression, dense extracellular matrix, immune suppression, and heterogeneous vascular perfusion [158,159]. Therefore, cancer self-monitoring nanomedicines should not only visualize tumor accumulation but also provide information on intratumoral distribution, payload release, therapeutic activation, and early biological response. A central role of self-monitoring cancer nanomedicine is to clarify whether tumor accumulation leads to functional therapeutic delivery. In a personalized cancer medicine study, 100 nm liposomes containing anticancer drugs and synthetic DNA barcodes were used to evaluate drug activity within triple-negative breast cancer tumors [160]. The barcoded nanoparticles accumulated in tumors, entered tumor cells, and allowed the activity of different drugs to be analyzed at the single-cell level after barcode recovery. Importantly, the barcode-based diagnostic screen predicted that gemcitabine would be the most effective treatment, and subsequent therapy mirrored this prediction in tumor-bearing mice [160]. This study illustrates how cancer theranostics can move beyond passive imaging of accumulation toward treatment selection based on tumor-level nanomedicine performance. Tumor microenvironment-responsive systems are particularly suitable for cancer self-monitoring because the same pathological features that trigger therapy can also activate imaging signals. A dual-stimuli-responsive nanoprobe based on asymmetric cyanine and glycosyl-functionalized gold nanorods was designed to respond synergistically to acidic pH and tumor-associated matrix metalloproteinases [161]. The nanoprobe remained largely silent under non-tumor conditions but generated near-infrared fluorescence in the tumor microenvironment, helping reduce background signals and false-positive activation. The activated signal identified the tumor site and guided photothermal therapy, with tumor temperature reaching approximately 50 °C within 6 min after 808 nm laser irradiation at 4 h post-injection [161]. This example demonstrates how tumor-specific activation can be used to connect imaging-guided localization with therapeutic timing. The nanoprobe design and its dual MMP/pH-responsive fluorescence activation and photothermal mechanism are illustrated in Figure 5.

Self-monitoring cancer nanomedicine is also useful for addressing multiple biological barriers simultaneously. A dual size- and charge-transformable “Trojan Horse” nanotheranostic system was developed to overcome circulation, tumor penetration, cellular uptake, and intracellular drug release barriers [162]. The particles remained PEGylated and relatively stable during circulation but transformed in the acidic tumor microenvironment to release smaller, more positively charged active pharmaceutical ingredient-based nanotheranostics. This transformation improved tumor penetration and cellular internalization, while near-infrared fluorescence imaging visualized biodistribution and MRI monitored tumor accumulation and therapeutic effects [162]. Such transformable systems show how disease-responsive design can integrate delivery monitoring, microenvironment-triggered activation, and therapeutic feedback in one platform. Cancer self-monitoring is especially important for externally activated therapies, including photothermal and photodynamic therapy. A FRET-based dual-modal theranostic nanoprobe was developed for in situ visualization of cancer photothermal therapy [163]. This system showed high FRET efficiency of 88.2%, clear tumor visualization by near-infrared fluorescence and photoacoustic imaging, and a tumor signal-to-background ratio of 14.5 at 24 h after injection [163]. During photothermal therapy, heat-induced disruption decreased FRET efficiency and produced fluorescence and photoacoustic signal changes, enabling in situ visualization of the therapeutic process. This type of self-feedback system is valuable because photothermal therapy depends strongly on local nanoparticle concentration, laser timing, tissue depth, and heat distribution. Response monitoring is another important function of cancer self-monitoring nanomedicine. A near-infrared organic afterglow nanoreporter was designed to visualize self-generated reactive oxygen species during photodynamic-mediated immunogenic cell death [164]. The semiconducting polymer-based nanoparticles emitted long-wavelength afterglow signals above 800 nm and showed a relationship between afterglow intensity, ROS generation, immunogenic cell death, and treatment outcome [164]. Because immunogenic cell death can promote dendritic cell activation and antitumor immune responses, this strategy provides a useful example of how cancer nanotheranostics can monitor not only therapeutic activation but also downstream immune-related response. Taken together, cancer applications highlight the main value of self-monitoring theranostic nanomedicine: the ability to separate and connect multiple stages of therapy, including tumor accumulation, penetration, payload release, therapeutic activation, and biological response. Tumor-associated signals such as acidic pH, protease activity, hypoxia, glutathione, ROS, and immune remodeling can be used to design disease-responsive imaging readouts. However, tumor signals must be interpreted carefully because a strong imaging signal does not necessarily indicate homogeneous distribution, intracellular delivery, effective payload release, or durable therapeutic response. Therefore, cancer self-monitoring nanomedicines should ideally combine mechanistically defined signal activation with quantitative validation, histological analysis, and therapeutic outcome assessment. This approach may support adaptive treatment timing, personalized combination therapy, and more precise interpretation of nanomedicine performance in heterogeneous tumors.

### 6.2. Broader Applications: Inflammatory and Immune-Related Diseases

**Figure 6:**
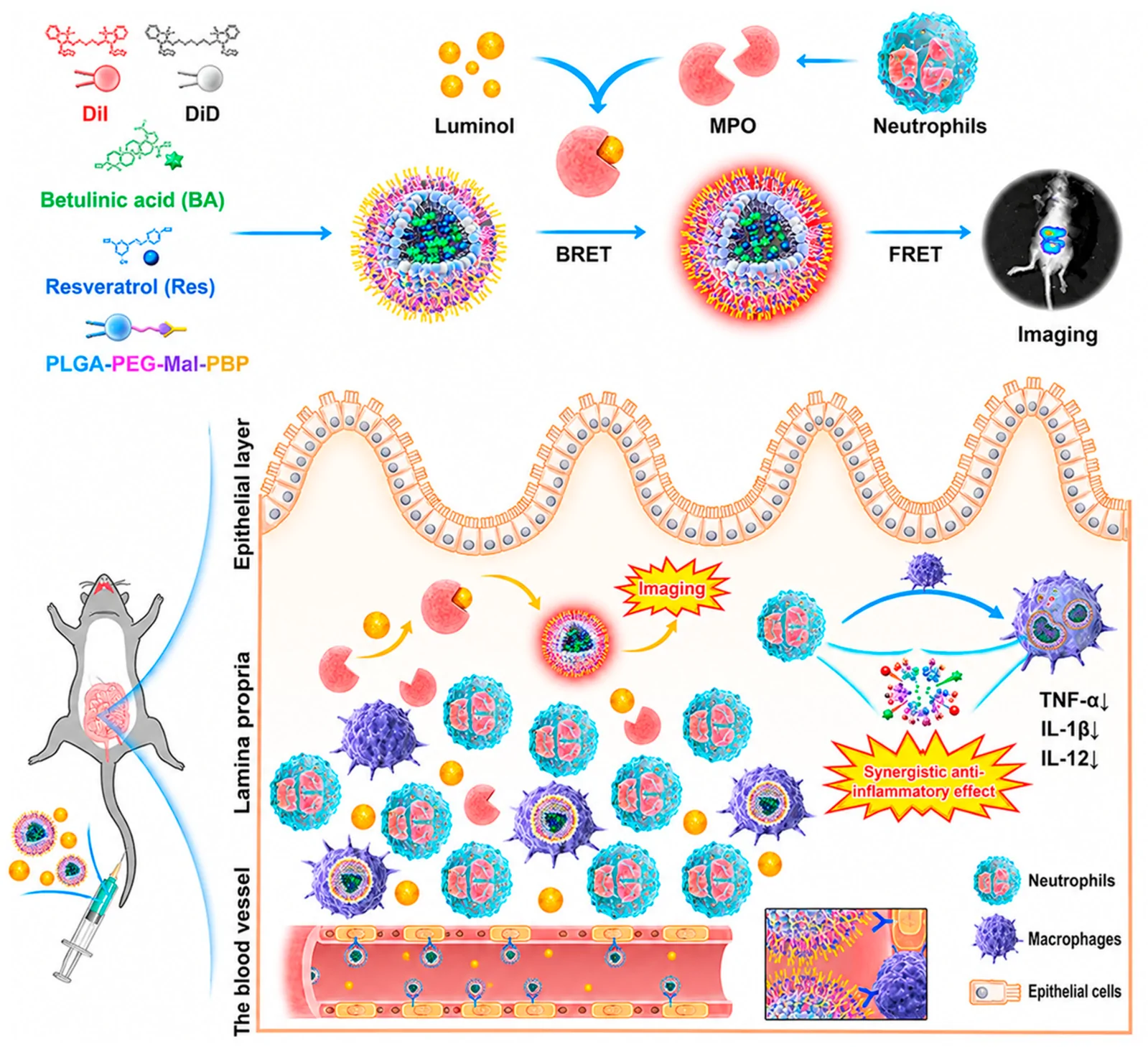
MPO-responsive BRET/FRET nanoplatform for targeted imaging and combination therapy of ulcerative colitis. Betulinic acid (BA) and resveratrol (Res), together with the FRET dyes DiL and DiD, were encapsulated in PBP-functionalized PLGA nanoparticles. At inflamed colonic sites, neutrophil-associated myeloperoxidase (MPO) catalyzes luminol chemiluminescence, initiating sequential BRET and FRET to generate red-shifted fluorescence for inflammation imaging. Because signal generation depends on MPO activity, the resulting optical signal provides a functional readout of neutrophil-associated inflammatory activity rather than nanoparticle localization alone. Targeted nanoparticle accumulation and the combined delivery of BA and Res suppress inflammatory-cell activation and pro-inflammatory cytokine production. Adapted from Ref. [169] under the terms of the Creative Commons Attribution 4.0 International License (CC BY 4.0).

Inflammatory and immune-related diseases are important application areas for self-monitoring theranostic nanomedicine because inflammatory activity changes dynamically across tissues and over time. Rheumatoid arthritis (RA), inflammatory bowel disease (IBD), autoimmune disorders, atherosclerosis, chronic wounds, and acute tissue injuries commonly involve increased vascular permeability, infiltration of macrophages and neutrophils, excessive production of reactive oxygen species (ROS), inflammatory cytokine secretion, hypoxia, and overexpression of enzymes such as matrix metalloproteinases and myeloperoxidase (MPO) [165,166]. These molecular and cellular changes may occur before substantial anatomical abnormalities become detectable and may also decrease before tissue swelling or structural damage is completely resolved. Therefore, self-monitoring nanomedicines should not only visualize accumulation in inflammatory lesions but also provide information on local oxidative stress, enzyme activity, immune-cell recruitment, therapeutic exposure, and biological response. RA is a representative target because inflamed synovial tissues contain leaky neovasculature, activated macrophages, elevated ROS levels, hypoxia, and abnormal extracellular matrix remodeling [167]. An albumin–ceria nanotheranostic platform was developed to combine optical and optoacoustic imaging with intrinsic antioxidant therapy [168]. Following systemic administration, the nanoparticles accumulated in arthritic joints through interactions involving secreted protein acidic and rich in cysteine in synovial tissues, allowing their biodistribution to be tracked noninvasively. The ceria component scavenged excessive ROS, generated oxygen, reduced synovial hypoxia, and restored the imbalance between pro-inflammatory and anti-inflammatory macrophage phenotypes. These effects reduced inflammation and joint damage in experimental arthritis. This system demonstrates how imaging can confirm successful delivery to diseased joints while the nanomaterial simultaneously modifies the inflammatory microenvironment. However, because the optical and optoacoustic signals primarily represent nanoparticle localization, additional activity-responsive signals would be required to directly quantify ROS reduction or macrophage reprogramming. Inflammatory microenvironment-responsive imaging has also been incorporated into the treatment of ulcerative colitis. A P-selectin-targeted polymeric nanoplatform was constructed using approximately 164 nm poly(lactic-co-glycolic acid) nanoparticles containing the fluorescent dyes DiL and DiD and co-loaded with betulinic acid and resveratrol [169]. P-selectin-binding peptides enhanced nanoparticle localization in inflamed colonic tissues, while intravenously administered luminol reacted with MPO-associated oxidative species to generate bioluminescence. The emitted energy was sequentially transferred through bioluminescence resonance energy transfer and Förster resonance energy transfer, producing an amplified optical signal from inflammatory lesions. The nanoplatform preferentially accumulated in the inflamed colon, alleviated colitis symptoms, and maintained intestinal microbiota homeostasis without producing detectable organ injury [169]. Because signal generation depended on MPO activity rather than fluorescence from the carrier alone, this system provided functional information associated with neutrophil-mediated inflammation. Nevertheless, the signal could also be influenced by nanoparticle concentration and luminol availability, which should be considered when interpreting longitudinal changes. The nanoparticle design, MPO-dependent BRET/FRET signal generation, and anti-inflammatory mechanism are illustrated in Figure 6.

Self-monitoring strategies can also provide pharmacokinetic information on local biological drug delivery. Infliximab-loaded polyphenol–poloxamer nanoparticles were labeled with zirconium-89 and administered rectally for positron emission tomography-guided treatment of IBD [170]. PET imaging showed that the nanoparticles reached the colon, remained at inflamed sites for prolonged periods, and produced substantially lower systemic exposure than intravenously administered infliximab. Despite the localized administration, the nanoparticle formulation produced anti-inflammatory effects comparable to those of systemic infliximab treatment [170]. This approach demonstrates how quantitative imaging can confirm local drug deposition, retention, and reduced off-target distribution, thereby supporting optimization of the administration route and dosing interval. However, the radioactive signal represented infliximab-associated nanoparticle localization rather than direct suppression of tumor necrosis factor-α signaling, indicating that it functions mainly as a pharmacokinetic self-monitoring system. More direct coupling between inflammatory activity, signal generation, and therapy was achieved using hydrogen peroxide-responsive polyoxalate nanoparticles [171]. These nanoparticles reacted with hydrogen peroxide in injured tissues to generate carbon dioxide bubbles that could be detected by ultrasonography. The ultrasound signal increased with hydrogen peroxide concentration, while the same reaction consumed hydrogen peroxide and reduced oxidative stress. In rat models of skeletal muscle and Achilles tendon injury, local administration produced strong ultrasound contrast at the injured sites, with detectable signal enhancement lasting for several hours. Treatment also suppressed the expression of inducible nitric oxide synthase, interleukin-1β, and tumor necrosis factor-α, reduced macrophage infiltration and apoptosis, and improved tissue structure [171]. Because the inflammatory mediator responsible for signal generation was simultaneously consumed during treatment, this platform established a direct mechanistic connection between oxidative stress, imaging output, and anti-inflammatory activity. A progressive decrease in ultrasound signal could therefore indicate reduced hydrogen peroxide levels, although nanoparticle degradation and local clearance may also contribute to signal attenuation. Extracellular nucleic acids released from damaged or necrotic cells provide another target for integrated inflammatory imaging and treatment. A fluorescent nanoprobe was designed by conjugating thiazole orange to a 40 kDa dextran carrier, producing a multivalent nucleic acid-binding system with nanomolar affinity for mammalian and bacterial DNA and RNA [172]. Binding to extracellular nucleic acids activated fluorescence and simultaneously reduced their ability to stimulate inflammatory cytokine production by macrophages. In a mouse model of myocardial ischemia–reperfusion injury, the nanoprobe accumulated in injured myocardial tissue and enabled fluorescence-based identification of the lesion. Treatment decreased myocardial macrophage infiltration at 24 h and reduced infarct size to approximately 18 ± 9% on day 7 [172]. This system represents a particularly integrated form of self-monitoring theranostics because the detected pro-inflammatory biomarker was also directly neutralized by the nanoprobe. Changes in fluorescence could therefore provide information on both the initial burden of extracellular nucleic acids and their progressive sequestration during treatment. Taken together, inflammatory and immune-related diseases demonstrate the potential of self-monitoring nanomedicine to distinguish active molecular inflammation from residual anatomical damage. Signals generated by ROS, MPO, extracellular nucleic acids, or activated immune cells may provide a more functional assessment than persistent joint swelling, intestinal wall thickening, or injured tissue morphology. However, a strong imaging signal does not necessarily indicate severe inflammation because signal intensity may also depend on nanoparticle concentration, vascular permeability, local perfusion, activation efficiency, tissue penetration, and clearance. Self-monitoring inflammatory nanomedicines should therefore ideally separate signals corresponding to lesion accumulation, microenvironmental activation, and biological response. Combining longitudinal imaging with cytokine profiling, immune-cell phenotyping, and histological analysis may support adaptive dosing and more precise treatment of spatially and temporally heterogeneous inflammatory diseases.

### 6.3. Broader Applications: Infectious Diseases

**Figure 7:**
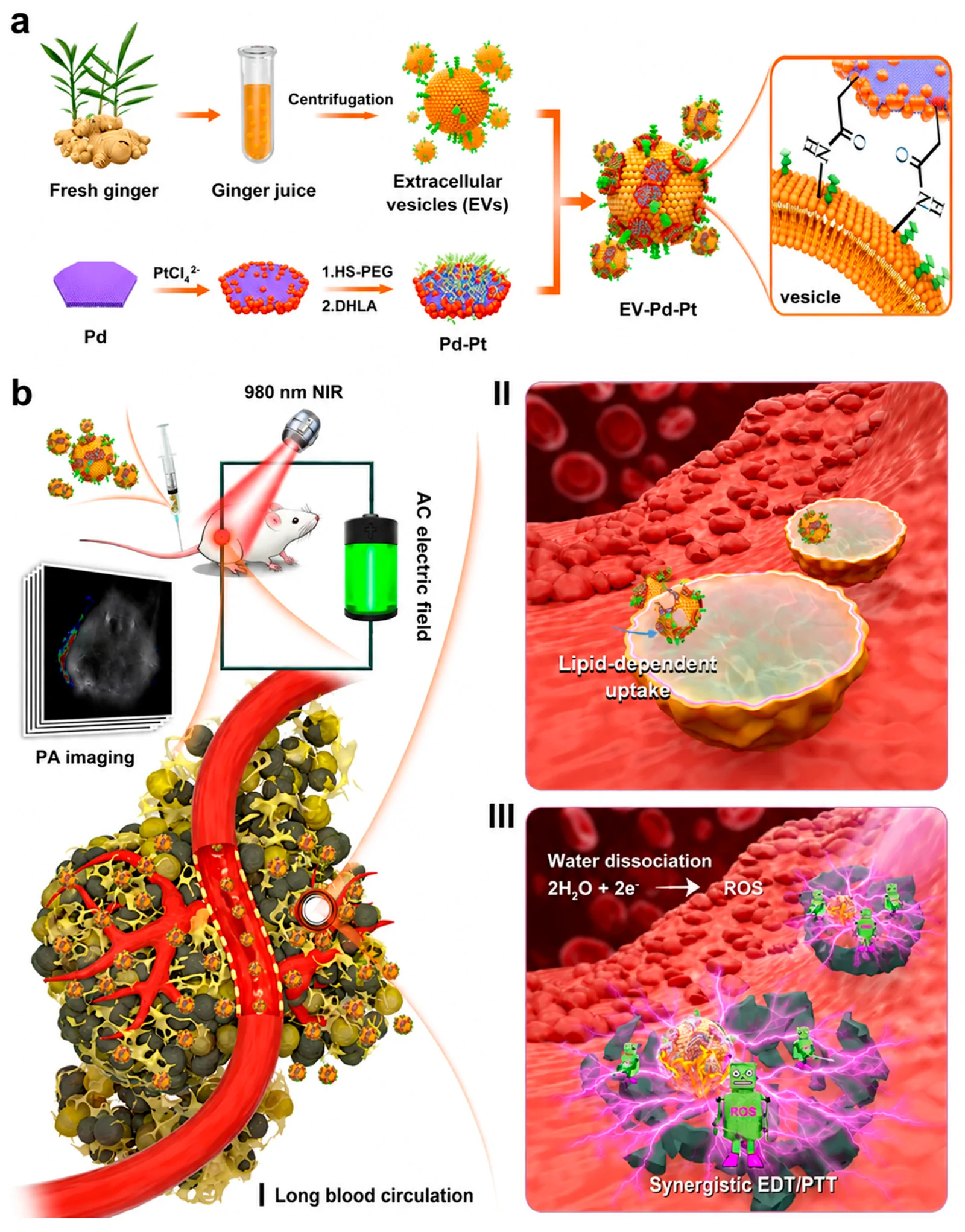
Ginger-derived extracellular vesicle-coated Pd–Pt nanoparticles for photoacoustic imaging-guided antibacterial therapy. (**a**) Preparation of EV–Pd–Pt nanoparticles by conjugating carboxyl-functionalized Pd–Pt nanoparticles to amino-rich ginger-derived extracellular vesicles. (**b**) (**I**–**III**) Following intravenous administration, the biomimetic nanoparticles exhibit prolonged circulation, preferential accumulation at infected sites, and lipid-dependent bacterial uptake. Combined application of an electric field and near-infrared irradiation induces synergistic electrodynamic and photothermal therapy for bacterial eradication. Adapted from Ref. [175] under the terms of the Creative Commons Attribution 4.0 International License (CC BY 4.0).

Infectious diseases are emerging as important targets for self-monitoring theranostic nanomedicine because conventional antimicrobial treatment often provides limited information on pathogen-site delivery, antimicrobial activation, and therapeutic response. These limitations are particularly important in multidrug-resistant infections, biofilm-associated infections, intracellular bacterial infections, and chronically infected wounds, where inadequate drug penetration and pathogen persistence can lead to treatment failure [173,174]. Infection sites commonly exhibit acidic pH, hypoxia, bacterial enzymes, pore-forming toxins, reactive oxygen species, immune-cell infiltration, and extracellular polymeric substances. These pathological features can be exploited to activate imaging signals, trigger antimicrobial release, or enhance photothermal and photodynamic treatment [173,174]. Therefore, self-monitoring infectious-disease nanomedicines should ideally distinguish pathogen-specific activity from nonspecific inflammation while providing information on lesion localization, biofilm penetration, intracellular delivery, antimicrobial activation, and bacterial clearance. A major role of self-monitoring infectious-disease nanomedicine is to determine whether accumulation at an infected site results in functional interaction with bacteria. A biomimetic platform composed of palladium–platinum nanosheets incorporated into ginger-derived extracellular vesicles was developed for multimodal imaging and synergistic electrodynamic–photothermal therapy [175]. The extracellular-vesicle membrane prolonged blood circulation and promoted lipid-dependent uptake of the nanoparticles by both *Staphylococcus aureus* and *Escherichia coli*. Following intravenous administration in mice with *S. aureus* abscesses, fluorescence and photoacoustic imaging showed time-dependent accumulation at the infected site, with maximum signals at approximately 8 h. Infrared thermal imaging further confirmed local photothermal activation, with the infected tissue reaching approximately 53 °C during 980 nm irradiation [175]. The platform generated hydroxyl radicals under an alternating electric field and combined this electrodynamic effect with photothermal treatment to disrupt biofilms and kill bacteria. Bioluminescence imaging of luciferase-expressing bacteria showed an almost complete loss of infection-associated signals after the combined treatment, providing direct longitudinal evidence of bacterial clearance. This system demonstrates how separate imaging signals can monitor nanoparticle accumulation, treatment activation, and antimicrobial response. The preparation, infection-site delivery, and combined electrodynamic–photothermal antibacterial mechanism of this platform are illustrated in Figure 7.

Infection-specific microenvironmental signals can also be used to connect drug release with bacterial physiology. A lactose-modified azocalix[4]arene carrier was developed for the treatment of multidrug-resistant *Pseudomonas aeruginosa*-infected diabetic wounds [176]. Multivalent lactose groups promoted binding to bacterial surface lectins and inhibited biofilm development, while azo linkages were cleaved by bacterial azoreductases under hypoxic conditions. Loading ciprofloxacin into the carrier therefore enabled bacterial targeting followed by hypoxia-responsive antibiotic release. A co-loaded fluorescent dye remained quenched within the carrier but produced increased fluorescence following azoreductase-mediated disassembly, allowing intracellular delivery and payload release to be visualized [176]. Fluorescence microscopy confirmed carrier internalization into bacteria and showed substantially greater dye and antibiotic release under hypoxic than normoxic conditions. In infected diabetic wounds, the formulation markedly reduced viable bacterial numbers, inflammatory-cell infiltration, and ROS levels and promoted granulation, angiogenesis, and wound closure. Because bacterial recognition, hypoxia-responsive disassembly, fluorescence recovery, and antibiotic release were mechanistically linked, this platform illustrates how infection-specific activation can provide information beyond nonspecific accumulation in inflamed tissue. Self-monitoring is also valuable for externally activated antimicrobial therapy because imaging can guide the timing and location of treatment. A thermo-responsive drug-delivery nanotransporter containing imipenem and the near-infrared dye IR780 was developed for chemo-photothermal treatment of multidrug-resistant bacterial infections [177]. IR780 enabled fluorescence visualization of the nanotransporter at infected tissues and converted 808 nm light into heat. When the local temperature exceeded approximately 43 °C, the fatty-acid-based core underwent a phase transition and released imipenem. The generated heat simultaneously damaged bacterial membranes increased antibiotic penetration, and enhanced inhibition of bacterial cell-wall synthesis [177]. The combined treatment eradicated clinically isolated methicillin-resistant *S. aureus* and multidrug-resistant *E. coli* at a substantially lower imipenem dose than free antibiotic treatment and prevented local infection from progressing toward systemic sepsis. This system connects fluorescence-based localization, photothermal monitoring, triggered drug release, and antimicrobial treatment, although its imaging signal primarily indicates nanocarrier distribution rather than bacterial viability. Intracellular bacterial infections require additional monitoring because therapeutic agents must enter mammalian host cells, locate intracellular pathogens, and preserve the viability of uninfected cells. A multifunctional nanobioconjugate was constructed by attaching spherical nucleic acids and aggregation-induced emission photosensitizers to living MS2 bacteriophages [178]. The resulting phage–DNA–photosensitizer conjugates rapidly entered mammalian cells and selectively recognized intracellular bacteria through the inherent targeting properties of the bacteriophage. Bacterial binding produced a detectable fluorescence signal, allowing intracellular infection to be localized. Light irradiation subsequently activated the photosensitizer to generate cytotoxic ROS, while the bacteriophage simultaneously contributed pathogen-specific binding and bacterial lysis [178]. The combined process eliminated intracellular bacteria, restored the viability of infected host cells, and accelerated the healing of infected wounds in diabetic mice without obvious systemic toxicity. This approach demonstrates how pathogen recognition can be integrated with fluorescence imaging and photodynamic treatment to distinguish functional intracellular targeting from simple uptake by infected cells. Biofilm infections are particularly suitable for localized visual monitoring because their acidic and structurally heterogeneous microenvironment changes during treatment. A microneedle theranostic patch was fabricated by incorporating manganese-doped carbon dots into a methacrylated gelatin matrix [179]. The microneedles penetrated the biofilm barrier and responded to the acidic biofilm microenvironment through a visible fluorescence change from yellow to turquoise, enabling naked-eye assessment of infection. Under 808 nm irradiation, the manganese-doped carbon dots generated local hyperthermia and hydroxyl radicals, which disrupted extracellular polymeric substances, dispersed the biofilm matrix, and killed the released bacteria [179]. The patch reduced inflammatory responses while promoting collagen deposition, angiogenesis, and healing in biofilm-infected wounds. Unlike conventional fluorescent nanocarriers that mainly indicate material localization, this system used a microenvironment-dependent optical response to report biofilm-associated acidity and initiate treatment only when required. However, because acidic conditions may also occur in noninfectious wounds, additional bacterial enzyme- or toxin-responsive elements would improve pathogen specificity. Taken together, infectious-disease applications demonstrate that self-monitoring nanomedicine can connect several critical stages of antimicrobial treatment, including infection-site accumulation, pathogen recognition, biofilm penetration, intracellular delivery, triggered drug release, therapeutic activation, and bacterial clearance. Pathogen-associated enzymes, toxins, lectins, biofilm acidity, hypoxia, and bacterial bioluminescence provide more infection-specific information than vascular permeability, ROS, or immune-cell infiltration alone. Nevertheless, no single signal can fully define treatment success. Nanocarrier fluorescence may persist after bacterial elimination, microenvironmental pH may remain abnormal during tissue repair, and reductions in inflammatory signals may not indicate complete pathogen clearance. Future systems should therefore combine independent readouts of carrier localization, pathogen-specific activation, bacterial viability, and host-tissue recovery. Such designs may help guide antimicrobial dosing, reduce unnecessary antibiotic exposure, and identify recurrent or incompletely treated infections before clinical deterioration. Comparison of representative noncancer applications reveals distinct monitoring priorities between inflammatory and infectious diseases. In inflammatory diseases, platforms such as the albumin–ceria nanotheranostic system for rheumatoid arthritis and the MPO-responsive polymeric nanoplatform for ulcerative colitis primarily monitor host-derived microenvironmental changes, including ROS, hypoxia, and neutrophil-associated inflammatory activity [168,169]. In contrast, infectious-disease platforms, including the Pd–Pt/ginger-derived extracellular-vesicle system and the hypoxia/azoreductase-responsive antimicrobial carrier, additionally require pathogen-specific localization, triggered antimicrobial activation or release, and direct assessment of bacterial clearance [175,176]. Thus, inflammatory theranostics mainly emphasize dynamic host-response biomarkers, whereas infectious theranostics must distinguish pathogen-specific activity from sterile inflammation and verify pathogen eradication. These differences highlight the need to tailor self-monitoring signals and therapeutic readouts to disease-specific biological targets and treatment endpoints. Across cancer, inflammatory and immune-related diseases, and infectious diseases, the principal value of self-monitoring theranostic nanomedicine lies in converting biological and physicochemical changes into actionable information during treatment. The examples discussed in Part 6 show that successful disease-site accumulation alone is insufficient to establish therapeutic efficacy. Effective systems must clarify whether nanomedicines penetrate pathological tissues, release their payloads, activate the intended therapeutic mechanism, and produce an appropriate biological response. Although the relevant biomarkers differ among tumors, inflammatory lesions, and infection sites, the same general design principle applies: imaging signals should be mechanistically linked to a defined stage of therapeutic action and validated against independent biological outcomes. Integrating multimodal imaging, longitudinal signal analysis, histological assessment, and molecular biomarkers may ultimately enable adaptive treatment strategies in which drug selection, treatment timing, and dosing are adjusted according to real-time nanomedicine performance.

## 7. Translational Challenges and Future Perspectives

Despite substantial progress in self-monitoring theranostic nanomedicine, translation from preclinical proof-of-concept studies to routine clinical use remains limited. Self-monitoring nanomedicines are intrinsically more complex than conventional drug-delivery systems because they integrate therapeutic payloads, imaging probes, responsive linkers, targeting components, and signal-generating mechanisms within a single formulation [1,80,81,82,83]. Although this multifunctionality can provide real-time information on biodistribution, payload release, therapeutic activation, and biological response, each additional component may alter pharmacokinetics, biological interactions, manufacturing reproducibility, safety, and regulatory classification. Consequently, future development should focus not merely on increasing the number of functions but on establishing quantitatively reliable, clinically actionable, and manufacturable monitoring systems. One of the most important translational challenges is determining what an imaging signal actually represents. A strong fluorescence or photoacoustic signal may reflect nanoparticle accumulation, probe activation, payload release, local environmental changes, or delayed clearance of an imaging component. Similarly, MRI, PET, SPECT, CT, and ultrasound signals may indicate the distribution of a contrast agent without confirming that the intact nanocarrier or therapeutic payload is present at the same location [180,181,182]. The intended measurand should therefore be defined explicitly for every self-monitoring platform. Signals designed to report biodistribution should be distinguished from those reporting carrier disassembly, linker cleavage, intracellular delivery, therapeutic activation, or downstream biological response. Without this distinction, visually compelling images may provide limited mechanistic or clinical information. Quantitative validation should extend beyond demonstrating a concentration-dependent signal under simplified in vitro conditions. Signal calibration should be performed in physiologically relevant media containing serum proteins, cells, enzymes, variable pH, redox gradients, and tissue-mimicking optical or acoustic properties [180,181,182]. In vivo imaging should then be correlated with independent measurements of nanocarrier concentration, released drugs, pharmacodynamic biomarkers, histological changes, immune-cell phenotypes, apoptosis, pathogen burden, or therapeutic outcome. A companion PET nanoreporter study provides an important example of this principle because tumor uptake of a zirconium-89-labeled liposomal reporter was quantitatively correlated with intratumoral doxorubicin concentrations and subsequent treatment efficacy [110]. Such studies demonstrate that imaging becomes clinically meaningful when the signal is validated against the biological or pharmacological event that it is intended to predict. Ratiometric probes, internal reference signals, and multimodal imaging may improve quantitative reliability, but they do not automatically eliminate uncertainty. Ratiometric measurements can reduce the effects of probe concentration, excitation intensity, and heterogeneous light delivery, whereas multimodal imaging can combine sensitive molecular detection with anatomical localization. However, the individual channels must provide independent and mechanistically interpretable information. Combining two carrier-associated signals may improve lesion visualization but does not necessarily provide additional information on payload release or response. Future studies should therefore establish calibration curves, activation thresholds, response times, signal reversibility, limits of detection, and relationships between signal changes and therapeutic events. Negative controls that lack the responsive linker, therapeutic payload, or biological trigger are also essential for determining signal specificity. Standardized experimental reporting is required to compare self-monitoring performance across different nanoplatforms. Current studies vary substantially in nanoparticle dose, administration route, animal model, disease stage, imaging instrument, acquisition settings, time points, image-processing procedures, and normalization methods. Minimum information reporting frameworks for bio–nano research have emphasized the need to provide sufficient physicochemical, biological, and methodological information to support reproducibility and cross-study comparison [180]. For self-monitoring nanomedicines, reporting should include not only particle size, polydispersity, surface charge, morphology, loading efficiency, and release kinetics but also probe-to-carrier ratio, signal stability, activation mechanism, signal-to-background ratio, imaging parameters, calibration procedure, and the biological meaning assigned to each readout [180,181,182]. Data processing and image normalization procedures should also be reported transparently, particularly when small differences in acquisition settings can markedly alter apparent signal intensity. Safety, biodegradation, and long-term biological fate represent additional barriers. Imaging probes and signal-generating components can modify the biological identity of the nanomedicine and influence protein-corona formation, immune recognition, organ distribution, degradation, and clearance [43,117]. Persistent inorganic nanoparticles, heavy-metal-containing probes, radiolabels, and highly hydrophobic fluorophores may accumulate in the liver, spleen, kidney, or other organs. Released imaging components may also follow different clearance pathways from the intact nanocarrier, complicating both safety assessment and signal interpretation. These issues are particularly important for longitudinal monitoring or repeated treatment, where cumulative exposure may be greater than in a single-dose imaging study. Biodegradable and excretable systems are therefore preferable when they can provide adequate signal stability and therapeutic performance. Lipid-based carriers, biodegradable polymers, protein-based nanoparticles, small organic fluorophores, and renal-clearable ultrasmall nanomaterials may reduce concerns regarding long-term retention [81,82,122]. Nevertheless, degradation kinetics must be balanced carefully. Premature degradation can cause off-target payload release and loss of the monitoring signal, whereas excessive stability can prevent drug release and prolong organ retention. Self-monitoring systems may offer an advantage in this context if they are designed to distinguish intact-carrier signals from degradation-product or released-payload signals. Long-term studies should evaluate complete mass balance, organ retention, metabolite formation, renal and hepatobiliary elimination, immunogenicity, and repeated-dose toxicity rather than relying solely on short-term histological analysis. Manufacturing reproducibility and scale-up are particularly challenging because self-monitoring formulations frequently require multiple synthesis, conjugation, loading, purification, and activation steps [77,83,182]. Small variations in particle composition may affect not only size and drug loading but also quenching efficiency, signal activation thresholds, relaxivity, radionuclide stability, acoustic response, or photothermal conversion. Imaging-related properties should therefore be treated as critical quality attributes together with conventional pharmaceutical parameters. Batch release criteria may need to include signal intensity, activation kinetics, response specificity, photostability, radiochemical purity, contrast-generating capacity, and correlation between signal activation and payload release. Scalable manufacturing technologies can help reduce these sources of variability. A feedback-controlled parallel microfluidic system was developed to continuously manufacture multicomponent lipid–polymer nanoparticles while maintaining their physicochemical characteristics [183]. Parallelized swirling microvortex reactors achieved a production capacity of approximately 1.8 kg per day, demonstrating that microfluidic processes can be expanded beyond laboratory-scale preparation when flow distribution and operating pressure are actively controlled [183]. Although the study did not specifically manufacture self-monitoring nanomedicines, the same principles are applicable to formulations requiring reproducible incorporation of drugs, imaging agents, and surface components. Continuous manufacturing, in-line process monitoring, modular conjugation chemistry, and quality-by-design approaches may ultimately be more suitable than highly operator-dependent multistep batch preparation. Regulatory evaluation is complicated by the fact that self-monitoring nanomedicines may combine characteristics of drugs, biologics, imaging agents, medical devices, and combination products. The U.S. Food and Drug Administration emphasizes that nanomaterial-containing drug products are subject to the same fundamental requirements for quality, safety, and efficacy as other products, but their size-dependent physicochemical and biological properties may require additional characterization and product-specific assessment [184]. European regulatory frameworks similarly evaluate nanotechnology-based medicinal products through existing medicinal-product and medical-device pathways while recognizing the need for product-specific scientific guidance and early interaction with regulatory authorities [185]. For externally activated platforms, the nanomedicine and the light, ultrasound, magnetic-field, or radiation-delivery device may also need to be evaluated as an integrated therapeutic system. The intended clinical function of the monitoring signal will strongly influence the regulatory evidence required. A signal used only for exploratory pharmacokinetic tracking may require different validation from one used to select patients, determine dosing, initiate external irradiation, or discontinue treatment. Decision-support frameworks developed for nanotechnology-enabled health products illustrate how physicochemical characterization and immunotoxicological testing can be prioritized according to product properties and intended use [186]. For self-monitoring systems, similar frameworks should additionally consider signal accuracy, false-positive and false-negative activation, imaging-device compatibility, decision thresholds, and the consequences of an incorrect treatment recommendation. Early consultation with regulatory authorities may help developers determine whether the diagnostic component represents a companion diagnostic, an integral part of a combination product, or an exploratory biomarker. Clinical translation will also depend on compatibility with existing imaging infrastructure. Optical imaging is highly useful for activatable molecular monitoring but is most clinically practical for superficial lesions, image-guided surgery, endoscopy, and catheter-based applications. PET and SPECT provide sensitive and quantitative whole-body tracking, MRI offers deep-tissue anatomical and functional information, CT provides rapid anatomical localization, and ultrasound enables real-time bedside imaging and externally triggered delivery. Importantly, early clinical nanomedicine studies have already established practical foundations for this translation. Iodine-124-labeled ultrasmall silica nanoparticles enabled PET-based assessment of whole-body distribution, renal clearance, and tumor targeting in patients with metastatic melanoma [90]. Serial SPECT/CT imaging of rhenium-188-labeled PEGylated liposomes similarly provided patient-level pharmacokinetic, dosimetric, biodistribution, and safety information in a phase 0 study [91]. These systems did not directly report payload release, but they demonstrate that quantitative nanoparticle tracking can be incorporated into clinical workflows. Recent translational frameworks emphasize that successful clinical development of nanomedicines requires rigorous physicochemical characterization, clinically relevant exposure, reproducible preclinical performance, scalable manufacturing under appropriate quality standards, and early regulatory planning [187]. A recent review of nanotheranostic clinical translation likewise highlighted persistent technical, manufacturing, regulatory, and clinical-development challenges despite continued advances in integrated diagnostic and therapeutic platforms [188]. These considerations further support the development of simplified self-monitoring platforms with clearly defined and clinically actionable imaging readouts rather than maximizing multifunctionality alone. A practical translational strategy may therefore be to begin with one or two monitoring functions that answer clearly defined clinical questions. For example, confirmation of target-site accumulation could be used to select patients likely to benefit from a nanotherapy, while a second signal could determine whether payload release or therapeutic activation has occurred. More complex systems that simultaneously report circulation, tissue penetration, cellular uptake, release, immune activation, metabolism, and response may provide valuable mechanistic information in preclinical studies but face substantial manufacturing and regulatory burdens. Functional minimalism—using the fewest components necessary to produce an actionable readout—may offer a more realistic route to clinical application. Artificial intelligence and machine learning may further increase the value of self-monitoring nanomedicine by analyzing longitudinal, spatial, and multimodal imaging datasets [189,190]. These approaches could support automated lesion segmentation, image registration, signal normalization, pharmacokinetic modeling, extraction of response-associated imaging features, and integration with histological, genomic, and clinical data. Machine learning may also assist in optimizing nanoparticle composition, predicting nano–bio interactions, identifying formulations with favorable delivery profiles, and selecting imaging time points [190]. In theranostic applications, early imaging patterns could potentially be used to predict treatment response before measurable anatomical changes occur [189]. However, AI-assisted interpretation will be reliable only when it is based on sufficiently large, standardized, and biologically annotated datasets. Models trained using a single nanoparticle formulation, imaging instrument, animal strain, or disease model may not generalize to other systems or patients. External validation, prospective testing, uncertainty estimation, explainable model outputs, and transparent reporting of training data will therefore be necessary. AI should not be used to compensate for poorly defined signal mechanisms. Instead, it should analyze monitoring signals that have already been validated against known delivery, release, activation, or response events. Feedback-guided treatment represents a promising longer-term direction. Imaging could be used to initiate external treatment only after adequate nanomedicine accumulation, adjust irradiation or ultrasound parameters according to local activation, repeat dosing when delivery is insufficient, or change therapy when early response signals are unfavorable. Ultimately, closed-loop systems may integrate sensing, therapeutic activation, response assessment, and subsequent treatment adjustment. Nevertheless, fully autonomous closed-loop nanomedicine remains largely conceptual. Before such systems can be implemented, robust relationships must be established among imaging signals, biological states, decision thresholds, and patient outcomes. Overall, the clinical future of self-monitoring theranostic nanomedicine will depend on balancing functional sophistication with translational practicality. The central objective should not be to incorporate the greatest possible number of therapeutic and diagnostic components, but to generate reliable information that improves a specific treatment decision. Progress will require mechanistically defined signal activation, quantitative and orthogonal validation, standardized reporting, biodegradable and clearable materials, reproducible manufacturing, regulatory planning, and compatibility with clinically available imaging modalities. By addressing these requirements, self-monitoring nanomedicines may progress from visually impressive preclinical platforms toward clinically useful systems for patient selection, treatment guidance, adaptive dosing, and personalized precision therapy.

## 8. Conclusions

Self-monitoring theranostic nanomaterials represent an important evolution from conventional image-guided nanomedicine toward functionally informative platforms that integrate biomedical imaging with therapeutic delivery and response assessment. Rather than simply identifying where a nanocarrier accumulates, these systems are designed to provide real-time or longitudinal information on nanoparticle biodistribution, biological fate, payload release, therapeutic activation, and downstream biological response. This distinction is particularly important because target-site accumulation alone does not confirm successful tissue penetration, effective payload release, activation of the intended therapeutic mechanism, or a meaningful biological outcome.

Self-monitoring capability can be achieved through the integration of always-on imaging probes, activatable signals, ratiometric sensors, Förster resonance energy transfer, self-quenching and dequenching mechanisms, environment-responsive contrast agents, and multimodal imaging. These strategies enable different stages of nanomedicine performance to be visualized, ranging from systemic circulation and organ distribution to carrier disassembly, intracellular release, externally triggered therapy, gene or RNA activity, oxidative stress modulation, immune activation, apoptosis, and tissue recovery. Fluorescence, MRI, photoacoustic, nuclear, CT, and ultrasound imaging each provide distinct advantages, but their value ultimately depends on whether the measured signal is mechanistically linked to the biological or therapeutic event that it is intended to represent.

Among disease-specific applications, cancer theranostics particularly highlights the potential of self-monitoring nanomaterials because tumor imaging can be directly integrated with the assessment of intratumoral distribution, payload release, microenvironment-responsive activation, photothermal and photodynamic therapy, immune-related responses, and early therapeutic outcomes. Such capabilities are especially valuable in heterogeneous tumors, where nanoparticle accumulation does not necessarily translate into uniform intratumoral penetration or effective treatment. By distinguishing carrier localization from functional therapeutic delivery, self-monitoring nanomaterials may provide more informative guidance for treatment timing, therapeutic activation, response assessment, and personalized cancer therapy. At the same time, applications in inflammatory and immune-related diseases and infectious diseases demonstrate that these monitoring principles can be extended to other pathological environments characterized by dynamic molecular and cellular changes.

Despite substantial progress, the interpretation and quantitative reliability of monitoring signals remain major challenges. Signal intensity may be influenced by nanoparticle concentration, tissue depth, local perfusion, probe degradation, activation efficiency, and clearance and therefore may not directly correspond to the amount of released drug or the magnitude of therapeutic response. Self-monitoring signals should consequently be validated using independent measurements such as drug quantification, histological analysis, pharmacodynamic biomarkers, molecular assays, immune-cell phenotyping, and therapeutic outcomes. Ratiometric or internally referenced designs, standardized imaging protocols, and orthogonal validation will be important for improving quantitative reliability and enabling meaningful comparison among different nanomaterial platforms.

Future development should also balance functional sophistication with biological safety and translational practicality. Simplified nanotheranostic systems that provide one or two clearly interpretable and clinically actionable signals may offer greater translational potential than highly complex platforms containing numerous therapeutic and diagnostic components. Biodegradable and clearable materials, reproducible manufacturing processes, clinically compatible imaging modalities, and clearly defined regulatory functions will therefore be essential for advancing self-monitoring nanomaterials beyond preclinical demonstrations. Artificial intelligence and multimodal data analysis may further support signal interpretation, response prediction, and adaptive treatment planning, provided that the underlying imaging signals are biologically validated. Overall, self-monitoring theranostic nanomaterials provide a promising framework for integrating biomedical imaging with cancer therapy by revealing where nanomaterials travel, when and where therapeutic payloads are released, whether treatment mechanisms are activated, and how tumors respond. Continued advances in signal specificity, quantitative validation, material safety, manufacturing reproducibility, and clinical integration may ultimately enable the transition from conventional image-guided nanomedicine toward feedback-guided, adaptive, and personalized cancer theranostics.

## Figures and Tables

**Table 1:** Representative self-monitoring theranostic platforms across major and complementary imaging modalities.

Imaging Modality	Representative Carrier/Platform	Main Monitoring Role	Trigger/Signal Mechanism	Study Setting	Key Advantage	Main Limitation	Refs.
Optical/NIR fluorescence	Single-walled carbon nanotube NIR-II fluorophore	Circulation and organ distribution	Always-on NIR-II fluorescence	In vivo	High sensitivity and temporal resolution	Limited tissue penetration and quantitative accuracy	[87]
MRI	Hollow MnO_2_ nanoplatform with DOX/Ce6	Microenvironment-responsive activation and drug release	Acidic pH-induced MnO_2_ degradation and Mn^2+^ release	In vitro/in vivo	Deep-tissue imaging and high anatomical resolution	Relatively low sensitivity; contrast may not directly represent payload release	[88]
Photoacoustic imaging	Semiconducting polymer nanoparticles	Molecular sensing	ROS-responsive photoacoustic signal change	In vivo	Functional molecular imaging at greater depth than fluorescence	Depth- and light-fluence-dependent	[89]
PET/SPECT	^124^I-labeled silica nanoparticles/^188^Re-PEGylated liposomes	Whole-body biodistribution, pharmacokinetics, and dosimetry	Radiotracer-associated signal	Clinical	Highly sensitive and quantitative whole-body imaging	Ionizing radiation; radiolabel may not always represent the intact carrier	[90,91]
CT	RGD-Bi_2_S_3_@mesoporous silica nanoparticles loaded with DOX	Tumor localization and image-guided therapy	NIR-triggered drug release and photothermal activation	In vitro/in vivo	Rapid, high-resolution anatomical imaging	Relatively low functional sensitivity and ionizing radiation	[92]
Ultrasound	Lipid/PLGA phase-change nanodroplets containing PFP and DOX	Triggered drug release and acoustic monitoring	Low-intensity focused ultrasound-induced phase transition	In vitro/in vivo	Real-time, nonionizing, and externally controllable	Strongly dependent on acoustic parameters and carrier stability	[93]
MPI	Fe_3_O_4_ nanocluster@PLGA core–shell nanocomposite loaded with DOX	Quantitative drug-release monitoring	Mild acidic degradation of PLGA and Fe_3_O_4_ nanocluster disassembly	In vitro/in vivo	Background-free and quantitative signal	Limited anatomical information and specialized instrumentation	[11]
Bioluminescence/BRET/chemiluminescence	Dye-loaded nanobubble contrast agent	Inflammation-associated molecular imaging	MPO-dependent luminol emission followed by BRET/FRET	In vivo	Low excitation background and high sensitivity	Substrate/reaction dependent; limited deep-tissue light propagation	[94]
Raman/SERS	GO–Fe_3_O_4_@Au@Ag–MPBA–DOX nanocomposite	Real-time payload-release monitoring	Acidic pH-induced boronic ester cleavage and SERS signal recovery	In vitro/in vivo	High molecular specificity and multiplexing capability	Limited penetration and specialized instrumentation	[95]

## Data Availability

No new data were created or analyzed in this study. Data sharing is not applicable to this article.

## Abbreviations

The following abbreviations are used in this manuscript:

CT	Computed tomography
FRET	Förster resonance energy transfer
IBD	Inflammatory bowel disease
MRI	Magnetic resonance imaging
PET	Positron emission tomography
STING	Stimulator of interferon genes
SPECT	Single-photon emission computed tomography
